# Characterization of normative hand movements during two functional upper limb tasks

**DOI:** 10.1371/journal.pone.0199549

**Published:** 2018-06-21

**Authors:** Aïda M. Valevicius, Quinn A. Boser, Ewen B. Lavoie, Glyn S. Murgatroyd, Patrick M. Pilarski, Craig S. Chapman, Albert H. Vette, Jacqueline S. Hebert

**Affiliations:** 1 Department of Biomedical Engineering, Faculty of Medicine and Dentistry, University of Alberta, Edmonton, Alberta, Canada; 2 Faculty of Kinesiology, Sport, and Recreation, University of Alberta, Edmonton, Alberta, Canada; 3 Glenrose Rehabilitation Hospital, Alberta Health Services, Edmonton, Alberta, Canada; 4 Department of Medicine, Faculty of Medicine and Dentistry, University of Alberta, Edmonton, Alberta, Canada; 5 Department of Mechanical Engineering, Faculty of Engineering, University of Alberta, Edmonton, Alberta, Canada; Universite de Nantes, FRANCE

## Abstract

**Background:**

Dexterous hand function is crucial for completing activities of daily living (ADLs), which typically require precise hand-object interactions. Kinematic analyses of hand trajectory, hand velocity, and grip aperture provide valuable mechanistic insights into task performance, but there is a need for standardized tasks representative of ADLs that are amenable to motion capture and show consistent performance in non-disabled individuals. Our objective was to develop two standardized functional upper limb tasks and to quantitatively characterize the kinematics of normative hand movement.

**Methods:**

Twenty non-disabled participants were recruited to perform two tasks: the Pasta Box Task and Cup Transfer Task. A 12-camera motion capture system was used to collect kinematic data from which hand movement and grip aperture measures were calculated. Measures reported for reach-grasp and transport-release segments were hand distance travelled, hand trajectory variability, movement time, peak and percent-to-peak hand velocity, number of movement units, peak and percent-to-peak grip aperture, and percent-to-peak hand deceleration. A between-session repeatability analysis was conducted on 10 participants.

**Results:**

Movement times were longer for transport-release compared to reach-grasp for every movement. Hand and grip aperture measures had low variability, with 55 out of 63 measures showing good repeatability (ICC > 0.75). Cross-body movements in the Pasta Box Task had longer movement times and reduced percent-to-peak hand velocity values. The Cup Transfer Task showed decoupling of peak grip aperture and peak hand deceleration for all movements. Movements requiring the clearing of an obstacle while transporting an object displayed a double velocity peak and typically a longer deceleration phase.

**Discussion:**

Normative hand kinematics for two standardized functional tasks challenging various aspects of hand-object interactions important for ADLs showed excellent repeatability. The consistency in normative task performance across a variety of task demands shows promise as a potential outcome assessment for populations with upper limb impairment.

## Introduction

Dexterous hand function is essential for successfully performing many activities of daily living (ADLs). Neurological or musculoskeletal impairments such as stroke [1], spinal cord injury [2], and upper limb amputation [3,4] result in deficiencies in hand and upper limb function, such that alternate control strategies and compensations must be used to accomplish ADLs. A key aspect of ADLs are hand-object interactions, where successfully reaching, grasping, and transferring an object is crucial for task completion. Quantifying these hand-object interactions in non-disabled populations to allow comparison to strategies used by impaired individuals could provide a valuable tool for assessing hand function.

One method of examining hand-object interactions is through kinematic analysis using optical motion capture, either to measure joint angles of the full upper body kinematic chain to infer hand function, or to directly quantify hand function through specific features of how the hand moves [5]. However, the design of the object interaction task becomes crucial in developing a standard assessment protocol that is repeatable and reliable for comparison within a normative population and across impaired populations. Specifically, hand movement during object interactions is influenced by an object’s extrinsic (location and orientation) and intrinsic parameters (size, color, shape, mass, and texture) [6,7]. When reaching for objects, normative adult behaviour will show typical hand trajectories, hand velocities, and grip aperture motions [7–9]. Reaching is influenced by the object’s extrinsic parameters, and typically characterized by a straight or gently curved hand trajectory path from an initial hand position to the object [8], with a smooth bell-shaped velocity profile with one velocity peak occurring approximately halfway through the movement [9], and with greater peak hand velocities observed for targets that are further away [10]. Grasp is primarily influenced by intrinsic parameters [6] and characterized by hand pre-shaping at hand movement onset [7], with grip aperture (the distance between the thumb and index finger) reaching a maximum at approximately 60 to 70% of the reaching phase, followed by hand closing around the object [9]. Grip aperture is also a function of object size, where a larger grip aperture is required for larger objects [6,9].

Given the importance of object interactions in ADLs and the influence of an object’s intrinsic and extrinsic proprieties on hand movement, task selection for kinematic analysis should include ecologically valid tasks for clinical assessment. Although some upper limb kinematic assessment protocols for motion capture mimic functional movements, such as hand to head (e.g., for combing hair), hand to shoulder (e.g., for dressing, applying deodorant), and hand to back pocket (e.g., for reaching for wallet, perineal care) [11–15], they provide limited information on hand function during real object interactions. In clinical populations, reaching and grasping tasks using real objects have shown alterations in hand kinematics such as asymmetries in hand velocity profile and decoupling of reach and grasp in those with hemiparesis [1,16,17], spinal cord injury [2] and with use of a prosthesis [18]. These studies have used a variety of objects for grasping such as a cup [16], ball [1], or cylinder [1,17,18]. However, it has been shown that not only the object characteristics but also the goal of the object interaction will affect grasp kinematics [19]. Therefore, a standardized functional task protocol for kinematic assessment with applicability to clinical populations would ideally involve reaching and grasping real objects, with specific movement goals, in order to most accurately mimic typical daily tasks.

Clinical assessments currently exist that evaluate hand function involving hand-object interactions. Performance tests, such as the Jebsen Test of Hand Function [20], the Box and Blocks Test [21], and Standardized Object Test [22], as well as subjective rater tests, such as the Action Research Arm Test [23] and Assessment of Capacity of Myoelectric Control (ACMC) [24] are commonly administered in a clinical environment. Although these tests involve hand-object interactions and provide a global outcome measurement of function, they do not allow for precise quantitative assessment of grasp, dexterity, movement quality, and efficiency. Ideally, functional assessment tasks used for kinematic analysis would utilize elements of these current clinical assessments, such as moving the arm in different positions; lateral reaches; crossing the body’s midline; adjusting hand opening and closing; varying grasp patterns; modulating force when gripping; and grasping and releasing objects.

The objectives of this study were to develop two standardized functional upper limb tasks with hand-object interactions that result in consistent and repeatable performance in non-disabled individuals, and to use kinematic analysis to quantitatively characterize hand movement for those two tasks. The hypothesis was that the performance of the tasks in non-disabled individuals would result in consistent performance (low variability) within and across performers and good between-session repeatability due to the standardized sequencing of the task movements.

## Methods

### Functional task development

The tasks were developed through iteration and consensus by a team involving a movement neuroscientist, kinesiologist, physiatrist, and occupational therapist. Current best-practice outcome measures for upper limb function were explored for commonalities in task requirements [20,21,24–27]. Functional tasks incorporated elements that would be challenging, but not impossible for clinical populations to complete, while being representative of ‘real-world’ tasks that might be performed in anyone’s daily environment. A key feature of the tasks was that they needed to be amenable to motion capture with standardized, discrete movement sequences to optimize kinematic analysis of hand movement characteristics across performers. Specifically, standardization of order of task execution allows kinematic data segmentation into specific movement sequences that can be averaged across trials and individuals to isolate characteristic movement strategies.

**Fig 1:**
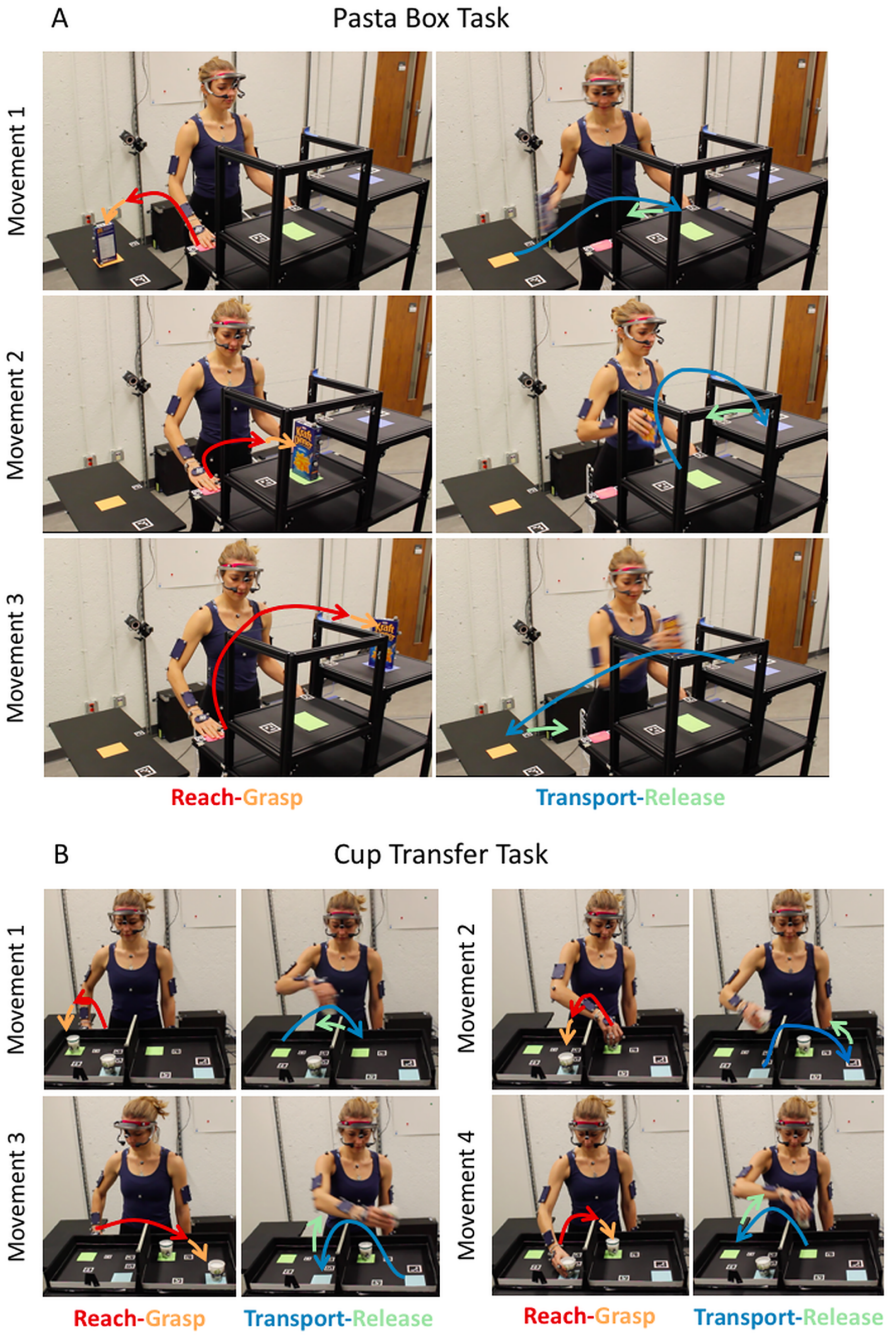
Functional tasks. Sequence of the Pasta Box Task (A) and Cup Transfer Task (B). During the Pasta Box Task, the participant moved the box of pasta from a lower side table to two shelves of different heights in front of them (Movements 1 and 2) and back again to the start position (Movement 3). Tasks were completed using three distinct movements, with standardized placement positions, including returning the hand to a standard “home” position at the end of each movement. During the Cup Transfer Task, the participant moved two compliant cups filled with therapeutic beads over a partition to a target location using a top grasp for the first cup and a side grasp for the second cup (Movements 1 and 2). These movements were followed by a return of the hand to the “home” position, and then by moving the cups back to their initial positions using respective types of grasps (Movements 3 and 4).

Two standardized functional tasks were developed, the Pasta Box Task and Cup Transfer Task (Fig 1). Full task set up and descriptions are available in the supplementary materials. The Pasta Box Task (S1 Text) was designed to mimic moving objects from a counter to a cupboard, between cupboards at different heights, and across the body’s midline. The Pasta Box Task consisted of three movements, during which the performer moved a box of pasta from a lower side shelf on their right (height: 30 inches) to a shelf in front of them (height: 43 inches); then to a second shelf at a higher height across the body (height: 48 inches); and then back to the starting position. The performer was required to return the hand to a “home” position after each specified movement. The Cup Transfer Task (S2 Text) was designed to involve greater risk by using compliant cups with content that can be spilled, and requiring careful placement around barriers such as would be encountered at a sink or countertop. The Cup Transfer Task consisted of four movements, where the performer moved two compliant cups filled with therapeutic beads from an initial position on the right side of a box to specific target positions on the left side of the box, and then back again to the start locations, while having to clear a middle partition. The box was placed at a standard counter height of 36 inches to recreate a real-world environment. The performer was required to return the hand to a “home” position after the first two movements and at the end of the task. In order to challenge grasp capabilities which might be difficult for impaired populations, two types of grasps, linked to the placement of the cups in the box, were required: a top grasp for the near cup and a side grasp for the far cup.

### Study participants

Twenty non-disabled individuals (11 male; 18 right-handed; age: 25.8 ± 7.2 years; height: 173.8 ± 8.3 cm; mean ± standard deviation) were recruited to participate in the study. They had no upper body pathology or history of neurological or musculoskeletal injuries within the past two years. The study followed the Declaration of Helsinki guidelines and was approved by the University of Alberta Health Research Ethics Board (Pro00054011), the Department of the Navy Human Research Protection Program (DON-HRPP), and the SSC-Pacific Human Research Protection Office (SSCPAC HRPO). The individual in Fig 1 has provided written consent to publish the photographs.

### Experimental setup and procedures

A 12-camera Vicon Bonita motion capture system (Vicon Motion Systems Ltd, Oxford, UK) with an accuracy of 0.5 mm and 0.5 degrees was used to collect three-dimensional marker trajectories at 120 Hz. A rigid plate with three 11.1 mm reflective markers was attached to the dorsal side of the right hand using double-sided tape. Two 14 mm single reflective markers were attached to the middle phalange of the index finger and the distal phalange of the thumb. Additional markers and marker clusters were attached to upper body segments (pelvis; trunk; upper arms; forearms; head), but were not used in the current analysis.

Prior to data collection, each participant received verbal instructions, a demonstration, and a practice trial to be familiarized with the tasks. Each participant performed a minimum of 20 successful trials for both tasks. If there was an error in performance of the task (i.e., dropping the object), that trial was marked as an “error trial” and the trial was repeated until a total of 20 error-free trials were recorded. The order of the tasks was block-randomized, with ten participants starting with the Pasta Box Task and ten with the Cup Transfer Task. Ten participants returned on a separate day for repeatability testing after the initial testing session (7.5 months ± 11 days), with the identical set up. The same two testers ran all data collection sessions.

### Experimental data analysis

Raw marker trajectory data were filtered using a second-order, low-pass Butterworth filter with a cut-off frequency of 6 Hz [28]. Motion capture data were segmented based on hand velocity, object velocity, and grip aperture (Table 1). The Pasta Box Task was divided into three movements, and the Cup Transfer Task was divided into four movements. Each task had a standard starting position for the hand (labelled ‘home’). Participants had to bring their hand to the “home” position after each movement for the Pasta Box Task and after the first two movements of the Cup Transfer Task to allow for task segmentation and standardization. Each movement was composed of four phases: reach, grasp, transport, and release. For the quantitative kinematic analysis, due to the short duration of grasp and release phases, and to interpret the results in light of functional hand movement sequences, the reach and grasp phases were combined into a reach-grasp segment, and the transport and release phases into a transport-release segment.

**Table 1:** Segmentation and phase definitions.

Phase Name	Start/End	Definition
Reach	**Start**: Hand leaves the home position	First occurrence of the hand exceeding the ‘Hand Velocity Threshold’ OR first occurrence of the hand exceeding the ‘Target Distance Threshold’, whichever happens first
Grasp	**Start**: Closing of grip aperture	First occurrence of the hand falling below the ‘Grasp Distance Threshold’
Transport	**Start**: Start of object movement	First occurrence of the object exceeding the ‘Object Velocity Threshold’ OR first occurrence of the object exceeding the ‘Target Distance Threshold’, whichever happens first
Release	**Start**: End of object movement	First occurrence of the object falling below the ‘Object Velocity Threshold’ OR first occurrence of the object distance falling below the ‘Target Distance Threshold’, whichever happens last
	**End**: End of grip aperture opening	Last occurrence of the hand before exceeding the ‘Release Distance Threshold’

Phase start and end definitions for the two functional tasks. Each task movement is separated into four phases: reach, grasp, transport, and release. The start and end of the phases are based on kinematic variables of hand velocity, object velocity, grip aperture, and hand-to-target or object-to-target distance. ‘Hand Velocity Threshold’ was defined as 5% of the peak hand velocity during the trial. ‘Object Velocity Threshold’ was defined as 5% of the peak object velocity during the trial. ‘Grasp Distance Threshold’ and ‘Release Distance Threshold’ were based on average occurrence of peak grip aperture prior to and following object movement, respectively. ‘Target Distance Threshold’ was defined as the location of the hand or object during a transition phase with respect to the target plus a tolerable distance of 70 mm.

Kinematic measures were selected based on commonly reported measures in assessments of individuals with upper limb impairments [5,29]. Hand movement was calculated using the average position of the three markers on the hand plate. Grip aperture was defined as the distance between the markers attached to the index and thumb. Analysed measures were movement time, hand distance travelled, hand trajectory variability, peak hand velocity, percent-to-peak hand velocity, number of movement units, peak grip aperture, percent-to-peak grip aperture, and percent-to-peak hand deceleration. Percent-to-peak measures were defined as the percent of time elapsed before the peak for a specific reach-grasp or transport-release segment. Hand trajectory variability was quantified as the maximum of the mean three-dimensional standard deviation at each time-normalized point. Number of movement units was defined as a local maximum velocity, or velocity peak [30–32] of the hand and was calculated by finding the zero-crossings in the hand acceleration profile where the signal switches from positive to negative. The hand trajectory, hand velocity, and grip aperture time series were time-normalized by segment (resampled to have 100 points per segment), averaged across trials and participants for each segment, and resampled across segments based on average segment length (with 1,000 points per overall trial).

### Statistical analysis

Statistical analysis was completed using the SPSS software (IBM Corporation, Armonk, NY, USA). For each task, hand trajectory variability, movement time, peak hand velocity and percent-to-peak hand velocity were analyzed using a two-way repeated-measures analysis of variance (ANOVA) examining effects of movement (3 for Pasta, 4 for Cups) and segment (reach-grasp, transport-release). Significant interactions (p < 0.05) were examined by conducting simple main effect one-way repeated-measures ANOVA’s of movement at each level of segment. Significant main or simple main effects (p < 0.05) were followed up by conducting all pairwise comparisons with Bonferroni correction. Normality was assessed using the Kolmogorov-Smirnov Test and sphericity was assessed through a Mauchly’s Test of Sphericity. In cases where the assumption of sphericity was not met, a Greenhouse-Geisser Correction was applied and reported. For peak grip aperture, percent-to-peak grip aperture, and percent-to-peak hand deceleration a one-way repeated-measures ANOVA, with Bonferroni corrected pairwise comparisons where significant (p < 0.05), was conducted to assess potential differences between movements for the reach-grasp segment only.

A between-session repeatability analysis was performed by calculating the intra-class correlation (ICC) for model (2,k), the standard error of measurement (SEM), and the minimal detectable change (MDC) [33] between the first and second session for ten participants. SEM was calculated based on the ICC analysis scores. The equation for SEM was:

$$SEM=SD\;\sqrt{1-ICC}$$ (1)

where SD is the standard deviation of all the participants in the first session. The MDC was calculated based on the SEM values and the 95% confidence interval. The equation for MDC was:

$$MDC=SEM\times 1.96\times \sqrt{2}$$ (2)

where 1.96 is the z score associated with the 95% confidence interval [34]. SEM and MDC scores were also represented as a percentage of the absolute average measurement value to indicate relative error. ICC, SEM, and MDC values were calculated for movement time, peak hand velocity, percent-to-peak hand velocity, peak grip aperture, percent-to-peak grip aperture, and percent-to-peak hand deceleration. ICC values above 0.90 were considered to indicate reasonable reliability for clinical measurements, above 0.75 indicated good repeatability, and below 0.75 indicated poor to moderate repeatability [33].

## Results

### Task performance

Overall performance time (from start to finish) for the Pasta Box Task was 8.84 ± 0.34 seconds, and for the Cup Transfer Task 10.60 ± 0.49 seconds. Error trials occurred at a rate of 4% for the Pasta Box Task, and 11% for the Cups Transfer Task. The most common errors were sequence hesitation (Pasta Box Task: 38% of errors, Cup Transfer Task: 54% of errors), hitting a partition/obstacle (Pasta Box Task: 31% of errors, Cup Transfer Task: 25% of errors), and incorrect grasp of the object (Pasta Box Task: 12% of errors, Cup Transfer Task: 16% of errors).

Movement times, hand distance travelled, and hand trajectory variability for each movement are listed in Tables 2 and 3 for the Pasta Box Task and Cup Transfer Task, respectively. Statistical results including post-hoc pairwise comparisons are presented in Tables 4 and 5 for the Pasta Box Task and Cup Transfer Task, respectively, and discussed below in the relevant sections per task.

**Table 2:** Pasta Box Task kinematic measures.

		**Hand distance** **travelled (mm)**		**Hand trajectory** **variability (mm)**		**Movement** **time (sec)**		**Peak hand** **velocity (mm/s)**		**Percent-to-peak** **hand velocity (%)**	
		*Mean ± SD*	*WPV*	*Mean ± SD*		*Mean ± SD*	*WPV*	*Mean ± SD*	*WPV*	*Mean ± SD*	*WPV*
**Mvmt 1**	RG	464 ± 25	21	17 ± 5		0.97 ± 0.15	0.07	1007 ± 125	71	42.5 ± 4.0	2.9
	TR	850 ± 27	14	19 ± 3		1.36 ± 0.16	0.07	1330 ± 140	69	33.1 ± 3.7	4.0
**Mvmt 2**	RG	478 ± 21	12	13 ± 3		0.68 ± 0.11	0.05	1204 ± 151	54	43.2 ± 6.5	3.8
	TR	740 ± 72	23	19 ± 4		1.45 ± 0.18	0.08	1035 ± 114	52	45.8 ± 5.7	8.5
**Mvmt 3**	RG	701 ± 21	14	19 ± 4		0.84 ± 0.15	0.05	1466 ± 197	74	38.8 ± 5.2	4.9
	TR	1069 ± 22	16	32 ± 6		1.68 ± 0.24	0.10	1470 ± 164	94	32.5 ± 3.8	3.6
		**Number of** **movement units**		**Peak grip** **aperture (mm)**		**Percent-to-peak** **grip aperture (%)**		**Percent-to-peak** **hand deceleration (%)**			
		*Mean ± SD*	*WPV*	*Mean ± SD*	*WPV*	*Mean ± SD*	*WPV*	*Mean ± SD*	*WPV*		
**Mvmt 1**	RG	1.2 ± 0.3	0.3	117 ± 7	3	71.6 ± 5.3	3.1	58.2 ± 8.5	11.0		
	TR	1.3 ± 0.3	0.5	-	-	-	-	-	-		
**Mvmt 2**	RG	1.1 ± 0.1	0.2	107 ± 7	3	78.0 ± 4.3	3.1	74.4 ± 8.0	5.3		
	TR	2.3 ± 0.3	0.4	-	-	-	-	-	-		
**Mvmt 3**	RG	1.2 ± 0.2	0.3	110 ± 6	3	79.0 ± 4.1	3.3	72.8 ± 7.8	4.9		
	TR	1.8 ± 0.5	0.9	-	-	-	-	-	-		

Pasta Box Task measures for hand distance travelled, hand trajectory variability, movement time, peak hand velocity, percent-to-peak hand velocity, number of movement units, peak grip aperture, percent-to-peak grip aperture, and percent-to-peak hand deceleration. Data are presented, for movements and segments separately, as group means and across-participant standard deviations (SD). Average within-participant variability (WPV) is also presented for each measure. Movements are: Movement 1 (Mvmt 1), Movement 2 (Mvmt 2), and Movement 3 (Mvmt 3); segments are: reach-grasp (RG) and transport-release (TR).

**Table 3:** Cup Transfer Task kinematic measures.

		**Hand distance** **travelled (mm)**		**Hand trajectory** **variability (mm)**		**Movement** **time (sec)**		**Peak hand** **velocity (mm/s)**		**Percent-to-peak** **hand velocity (%)**	
		*Mean ± SD*	*WPV*	*Mean ± SD*		*Mean ± SD*	*WPV*	*Mean ± SD*	*WPV*	*Mean ± SD*	*WPV*
**Mvmt 1**	RG	380 ± 44	22	16 ± 3		0.86 ± 0.13	0.07	818 ± 117	60	43.7 ± 7.7	6.0
	TR	608 ± 36	21	16 ± 2		1.32 ± 0.15	0.10	970 ± 79	47	21.1 ± 3.8	4.2
**Mvmt 2**	RG	445 ± 45	30	27 ± 7		0.75 ± 0.15	0.07	1050 ± 104	62	25.9 ± 7.7	6.7
	TR	635 ± 46	26	17 ± 4		1.47 ± 0.15	0.10	898 ± 73	46	39.9 ± 7.7	6.3
**Mvmt 3**	RG	851 ± 35	25	26 ± 6		1.10 ± 0.17	0.07	1435 ± 157	75	42.5 ± 5.6	6.3
	TR	673 ± 42	28	18 ± 3		1.46 ± 0.17	0.10	976 ± 53	48	25.1 ± 2.1	2.3
**Mvmt 4**	RG	426 ± 43	25	24 ± 6		0.63 ± 0.09	0.06	1041 ± 113	59	25.0 ± 7.2	7.5
	TR	622 ± 42	25	17 ± 3		1.36 ± 0.17	0.10	956 ± 66	47	23.8 ± 5.3	7.1
		**Number of** **movement units**		**Peak grip** **aperture (mm)**		**Percent-to-peak** **grip aperture (%)**		**Percent-to-peak** **hand deceleration (%)**			
		*Mean ± SD*	*WPV*	*Mean ± SD*	*WPV*	*Mean ± SD*	*WPV*	*Mean ± SD*	*WPV*		
**Mvmt 1**	RG	1.3 ± 0.3	0.4	99 ± 5	3	80.9 ± 5.1	4.5	69.7 ± 7.9	9.2		
	TR	2.5 ± 0.4	0.8	-	-	-	-	-			
**Mvmt 2**	RG	1.3 ± 0.3	0.4	114 ± 6	3	71.9 ± 5.9	6.0	49.8 ± 8.2	8.7		
	TR	2.5 ± 0.5	0.8	-	-	-	-	-	-		
**Mvmt 3**	RG	1.4 ± 0.3	0.4	113 ± 7	2	81.0 ± 3.7	4.5	60.6 ± 5.7	5.4		
	TR	2.2 ± 0.7	1.0	-	-	-	-	-	-		
**Mvmt 4**	RG	1.3 ± 0.3	0.4	118 ± 7	3	78.1 ± 6.9	5.1	68.4 ± 13.5	10.7		
	TR	2.5 ± 0.4	0.7	-	-	-	-	-	-		

Cup Transfer Task measures for hand distance travelled, hand trajectory variability, movement time, peak hand velocity, percent-to-peak hand velocity, number of movement units, peak grip aperture, percent-to-peak grip aperture, and percent-to-peak hand deceleration. Data are presented, for movements and segments separately, as group means and across-participant standard deviations (SD). Average within-participant variability (WPV) is also presented for each measure. Movements are: Movement 1 (Mvmt 1), Movement 2 (Mvmt 2), Movement 3 (Mvmt 3), and Movement 4 (Mvmt 4); segments are: reach-grasp (RG) and transport-release (TR).

**Table 4:** Pasta Box Task statistical analysis results.

**Movement time (s)**			Interaction: Mvmt x Segment F(2, 38) = 238.5**		
	Mvmt 1	Mvmt2	Mvmt 3	F (Mvmt effect)	Pairwise
Reach-grasp	0.97	0.68	0.84	(2, 38) = 255.9**	2 << 3 << 1
Transport-release	1.36	1.45	1.68	(2, 38) = 128.5**	1 << 2 << 3
**Hand trajectory variability (mm)**			Interaction: Mvmt x Segment F(2, 38) = 22.6**		
	Mvmt 1	Mvmt 2	Mvmt 3	F (Mvmt effect)	Pairwise
Reach-grasp	17	13	19	(1.4, 27.5) = 23.6**	2 << 1,3
Transport-release	19	19	32	(1.5, 28.7) = 63.1**	1 << 3; 2 << 3
**Peak hand velocity (mm/s)**			Interaction: Mvmt x Segment F(2, 38) = 154.3**		
	Mvmt 1	Mvmt 2	Mvmt 3	F (Mvmt effect)	Pairwise
Reach-grasp	1008	1204	1466	(1.4, 26.4) = 183.1**	1 << 2 << 3
Transport-release	1330	1035	1470	(2, 38) = 199.1**	2 << 1 << 3
**Percent-to-peak hand velocity (%)**			Interaction: Mvmt x Segment F(2, 38) = 22.2**		
	Mvmt 1	Mvmt 2	Mvmt 3	F (Mvmt effect)	Pairwise
Reach-grasp	42.4	43.2	38.8	(1.4, 26.2) = 6.9*	3 < 1; 3 << 2
Transport-release	33.1	45.8	32.5	(2, 38) = 80.4**	1 << 2; 3 << 2
**Peak grip aperture (mm)**					
	Mvmt 1	Mvmt 2	Mvmt 3	F (Mvmt effect)	Pairwise
Reach-grasp	117	107	109	(1.3, 25.2) = 92.7**	2 << 3 << 1
**Percent-to-peak grip aperture (%)**					
	Mvmt 1	Mvmt 2	Mvmt 3	F (Mvmt effect)	Pairwise
Reach-grasp	71.6	77.9	79.0	(1.2, 23.5) = 61.3**	1 << 2,3; 2 < 3
**Percent-to-peak hand deceleration (%)**					
	Mvmt 1	Mvmt 2	Mvmt 3	F (Mvmt effect)	Pairwise
Reach-grasp	58.2	74.4	72.8	(1.1, 20.9) = 58.0**	1 << 2,3; 3 < 2

Pasta Box Task results of the two-factor and one-factor repeated-measures analysis of variance (ANOVA). The results for the interaction of Movement and Segment for the two-factor repeated-measures ANOVA is reported for hand trajectory variability, movement time, peak hand velocity, and percent-to-peak hand velocity. The effect of movement for the one-way repeated measures ANOVA is reported for peak grip aperture, percent-to-peak grip aperture, and percent-to-peak-grip hand deceleration. The simple main effects ANOVA is reported for hand trajectory variability, movement time, peak hand velocity, and percent-to-peak hand velocity.

** indicates that the F-statistic was significant at p < 0.001;

* indicates that the F-statistic was significant at p < 0.05; << indicates that the pairwise comparison was significantly smaller at p < 0.001; < indicates that the pairwise comparison was significantly smaller at p < 0.05.

**Table 5:** Cup Transfer Task statistical analysis results.

**Movement time (s)**				Interaction: Mvmt x Segment F(3, 57) = 167.1**		
	Mvmt 1	Mvmt2	Mvmt 3	Mvmt 4	F (Mvmt effect)	Pairwise
Reach-grasp	0.86	0.75	1.10	0.63	(3, 57) = 273.2**	4 << 2 << 1 << 3
Transport-release	1.32	1.48	1.47	1.36	(2.5, 48.1) = 55.2**	1 << 2,3; 4 << 2,3
**Hand trajectory variability (mm)**				Interaction: Mvmt x Segment F(3, 57) = 10.2**		
	Mvmt 1	Mvmt2	Mvmt 3	Mvmt 4	F (Mvmt effect)	Pairwise
Reach-grasp	16	27	26	24	(3, 57) = 19.5**	1 << 2,3,4
Transport-release	16	17	18	17	(3, 57) = 4.42*	1 < 3
**Peak hand velocity (mm/s)**				Interaction: Mvmt x Segment F(3, 57) = 130.3**		
	Mvmt 1	Mvmt2	Mvmt 3	Mvmt 4	F (Mvmt effect)	Pairwise
Reach-grasp	818	1050	1435	1041	(3, 57) = 176.3**	1 << 2,3,4; 2 << 3; 4 << 3
Transport-release	970	897	976	956	(3, 57) = 18.5**	2 < 1; 2 << 3,4
**Percent-to-peak hand velocity (%)**				Interaction: Mvmt x Segment F(3, 57) = 132.0**		
	Mvmt 1	Mvmt2	Mvmt 3	Mvmt 4	F (Mvmt effect)	Pairwise
Reach-grasp	43.7	25.9	42.5	25.0	(3, 57) = 54.1**	2 << 1,3; 4 << 1,3
Transport-release	21.1	39.9	25.1	23.8	(1.8, 35.0) = 70.0**	1 << 2,3; 3 << 2; 4 << 2
**Peak grip aperture (mm)**						
	Mvmt 1	Mvmt2	Mvmt 3	Mvmt 4	F (Mvmt effect)	Pairwise
Reach-grasp	99	114	113	118	(3, 57) = 102.1**	1 << 2,3,4; 2 < 4; 3 < 4
**Percent-to-peak grip aperture (%)**						
	Mvmt 1	Mvmt2	Mvmt 3	Mvmt 4	F (Mvmt effect)	Pairwise
Reach-grasp	80.9	71.9	80.9	78.1	(3, 57) = 24.9**	2 << 1,3; 2 < 4
**Percent-to-peak hand deceleration (%)**						
	Mvmt 1	Mvmt2	Mvmt 3	Mvmt 4	F (Mvmt effect)	Pairwise
Reach-grasp	69.7	49.2	60.6	68.4	(2.1, 40.4) = 45.4**	2 << 1,3,4; 3 << 1; 3 < 4

Cup Transfer Task results of the two-factor and one-factor repeated-measures analysis of variance (ANOVA). The results for the interaction of Movement and Segment for the two-factor repeated-measures ANOVA is reported for hand trajectory variability, movement time, peak hand velocity, and percent-to-peak hand velocity. The effect of movement for the one-way repeated measures ANOVA is reported for peak grip aperture, percent-to-peak grip aperture, and percent-to-peak-grip hand deceleration. The simple main effects ANOVA is reported for hand trajectory variability, movement time, peak hand velocity, and percent-to-peak hand velocity.

** indicates that the F-statistic was significant at p < 0.001;

* indicates that the F-statistic was significant at p < 0.05; << indicates that the pairwise comparison was significantly smaller at p < 0.001; < indicates that the pairwise comparison was significantly smaller at p < 0.05.

Movement times for reach-grasp segments were significantly smaller than for transport-release segments (p < 0.001), even in the single case where transport distance was shorter than reach distance during Movement 3 in the Cup Transfer Task (Table 3). For the Pasta Box Task, post-hoc pairwise comparisons revealed that movement time for both reach-grasp and transport-release segments was significantly different between all individual movements (p < 0.001). For the Cup Transfer Task, post-hoc pairwise comparisons revealed that movement time for both reach-grasp and transport-release segments was significantly different between individual movements (p < 0.001), except for the transport-release segment of Movement 1 compared to Movement 4 (p = 0.079) and Movement 2 compared to Movement 3 (p = 1.000).

**Fig 2:**
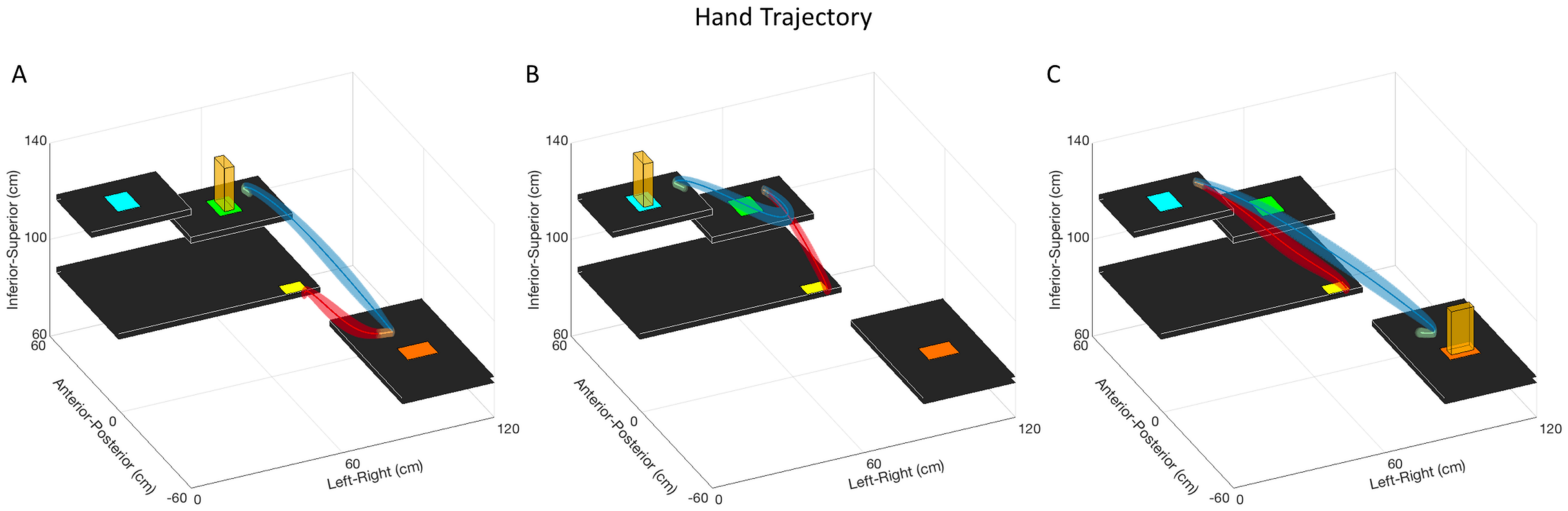
Pasta Box Task hand trajectory. Hand trajectories for the Pasta Box Task. The group average hand trajectory is plotted as a dark line, and the standard deviation of participant means as three-dimensional shading. Movement 1 (A), Movement 2 (B), and Movement 3 (C) are segmented into reach (red), grasp (orange), transport (blue), and release (green) phases. The maximum of the mean three-dimensional standard deviation was calculated for reach-grasp and transport-release segments in each movement to quantify variability, reported in Table 2.

**Fig 3:**
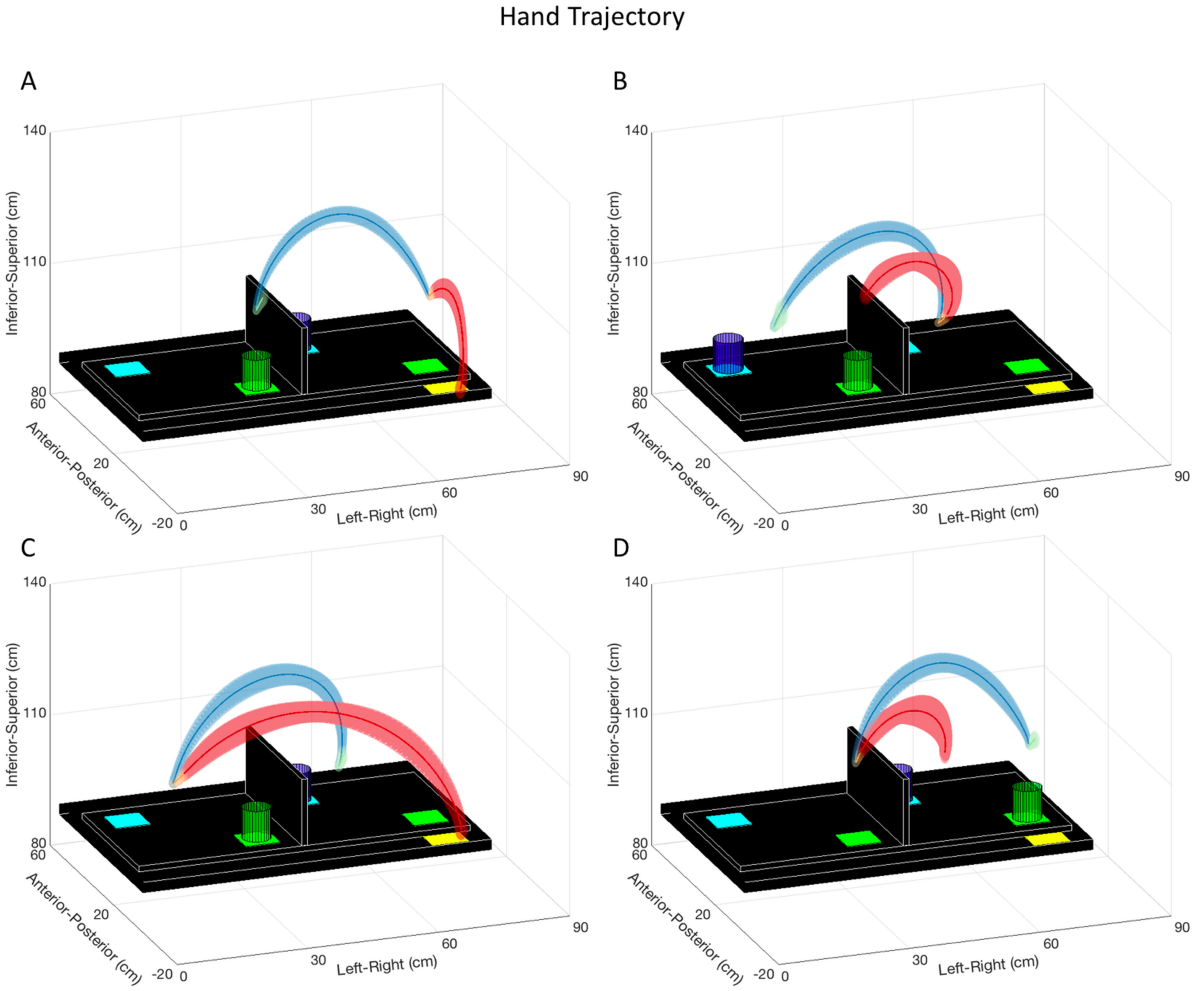
Cup Transfer Task hand trajectory. Hand trajectories for the Cup Transfer Task. The group average hand trajectory is plotted as a dark line, and the standard deviation of participant means as three-dimensional shading. Movement 1 (A), Movement 2 (B), Movement 3 (C), and Movement 4 (D) are segmented into reach (red), grasp (orange), transport (blue), and release (green) phases. The maximum of the mean three-dimensional standard deviation was calculated for reach-grasp and transport-release segments for each movement to quantify variability, reported in Table 3.

Hand trajectory graphs displaying the average hand trajectories and across-participant standard deviations are shown in Figs 2 and 3. Maximum hand trajectory variability was overall small for both tasks, ranging from 13 mm to 32 mm for the Pasta Box Task (Table 2) and 16 mm to 27 mm for the Cup Transfer Task (Table 3). For the Pasta Box Task (Table 4), during reach-grasp, Movement 2 was significantly less variable than Movement 1 and 3 (p < 0.001) and, during transport-release, Movement 3 was significantly more variable than Movement 1 and 2 (p < 0.001). For the Cup Transfer Task (Table 5), during reach-grasp, Movement 1 was significantly less variable than Movement 2, 3, and 4 (p < 0.001) and, during transport-release, Movement 1 was significantly less variable than Movement 3 (p < 0.05).

### Pasta Box Task

**Fig 4:**
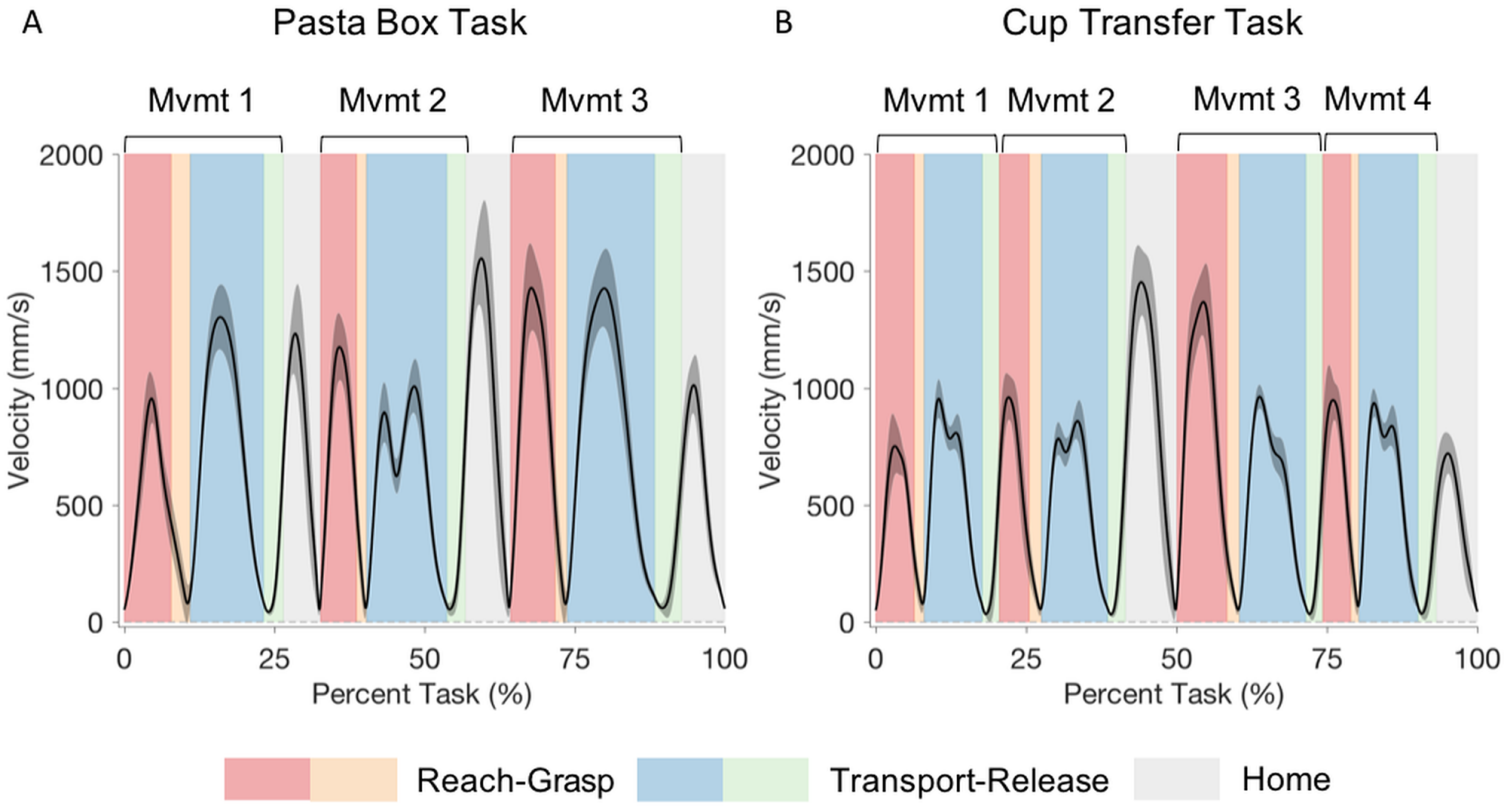
Hand velocity. Hand velocity graphs for Pasta Box Task (A) and Cup Transfer Task (B). The solid line represents the group average, and grey shading the standard deviation of participant means. The task is segmented into reach (red), grasp (orange), transport (blue), and release (green) phases for each movement, with light grey representing the return to “home” phase. Kinematics of the reach-grasp segment and the transport-release segment were analyzed together.

**Fig 5:**
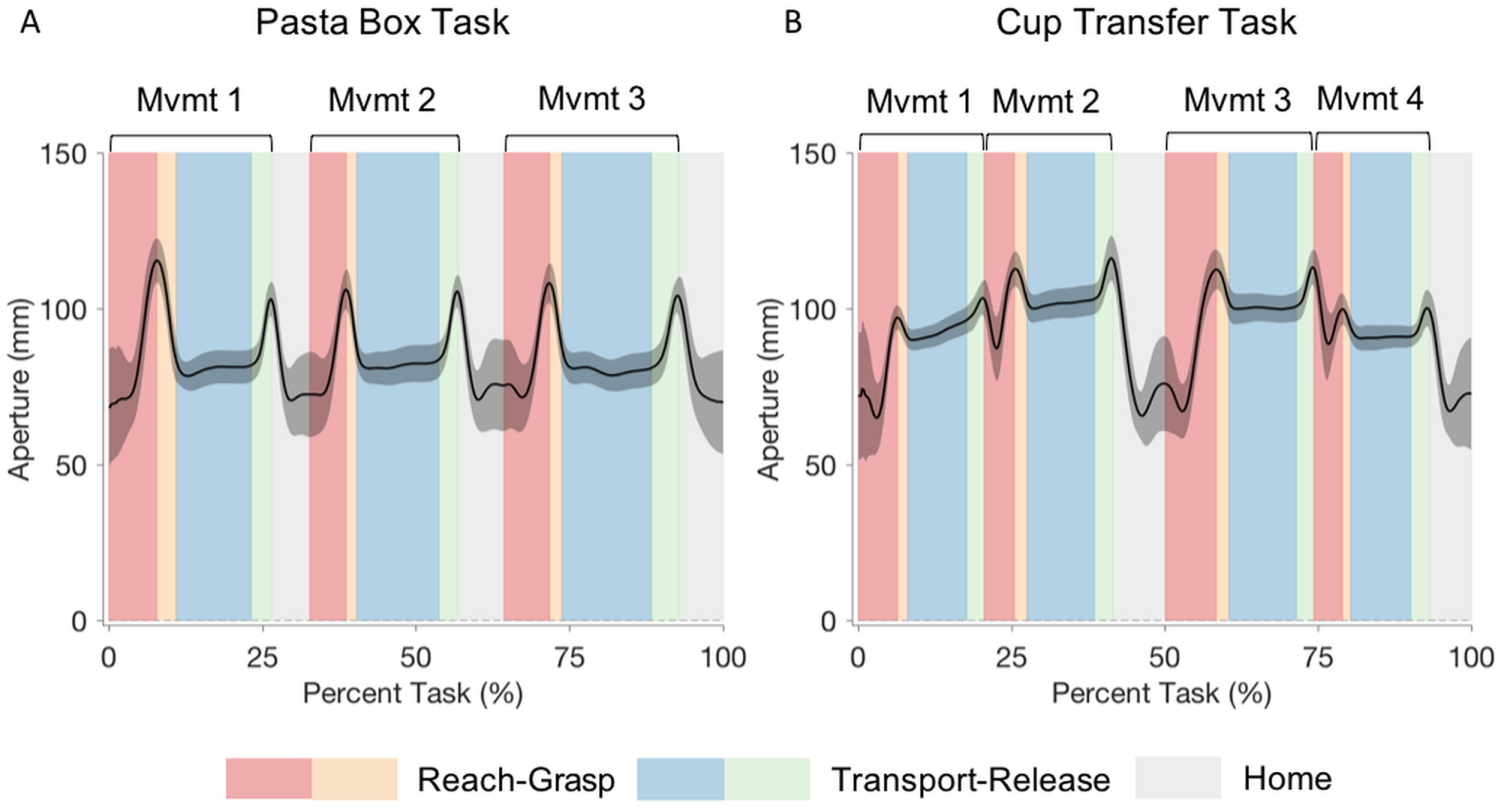
Grip aperture. Grip aperture graphs for Pasta Box Task (A) and Cup Transfer Task (B). The solid line represents the group average, and grey shading represents the standard deviation of participant means. The task is segmented into reach (red), grasp (orange), transport (blue), and release (green) phases for each movement, with light grey representing the return to “home” phase. Kinematics of the reach-grasp segment and the transport-release segment were analyzed together.

For the Pasta Box Task (Table 2), movements involving the side table location affected several kinematic parameters compared to other movements. Movement 1, where participants had to turn their body and reach to the side table to pick up the box, had the lowest peak hand velocity for the reach-grasp segment (Fig 4A), which was significantly different from Movements 2 and 3 (p < 0.001). Peak grip aperture (Fig 5A) was also greatest for the first reach-grasp segment, which was significantly different from the reach-grasp segment of Movements 2 and 3. The peak grip aperture during the first reach-grasp segment also occurred significantly earlier than for the following two movements (p < 0.001) and did not align with the percent-to-peak hand deceleration. The transport-release segments of Movement 1 and 3, both involving moving the box from or to the side cart, had similar percent-to-peak hand velocities (p = 1.000). They were significantly lower than for Movement 2 (p < 0.001).

The distinct feature of Movement 2 was that the participant had to transfer the box of pasta from the first shelf to the second shelf by moving around the middle cart barrier, which served as an obstacle. The transport-release segment of this movement had a significantly lower peak hand velocity than the other transport-release segments (p < 0.001), displayed two velocity peaks (indicated by the number of movement units in Table 2 and as seen in Fig 4A), and exhibited the greatest within-participant variability in percent-to-peak hand velocity.

Movement 3 had the longest distances for both reach and transport segments. Although the magnitude and timing of peak grip aperture for the reach-grasp segment was statistically different for each movement, the absolute values were very close. Reach-grasp of Movement 3 had significantly higher peak hand velocities than for reach-grasp in the other movements (p < 0.001), and the peak hand velocity in reach-grasp occurred significantly earlier than that of Movement 1 (p = 0.028) and Movement 2 (p < 0.001). Peak hand velocity during transport-release of Movement 3 was similarly significantly higher than that of Movement 1 and 2 (p < 0.001), but with a lower percent-to-peak hand velocity compared to Movement 2 (p < 0.001) (but not significantly different from Movement 1 (p = 1.000)).

### Cup Transfer Task

Overall, peak velocities for the Cup Transfer Task (Table 3) were significantly higher for reach-grasp segments than transport-release segments (p < 0.001). Slower movement during transport was expected given the risk of spilling the compliant cups filled with beads. As evidenced by the hand velocity graph (Fig 4B) and the number of movement units (Table 3), all transport-release segments displayed small, double hand velocity peaks, reflecting a consequence of transporting the cup over an obstacle in the vertical plane.

The Cup Transfer Task was unique in that it required two different grasp patterns, which we hypothesized would affect grip aperture and velocity of movements based on confidence in modulating grip patterns to not crush the cup and spill the contents. Movement 1 had the shortest distance to reach and the lowest peak velocity (Fig 4B) compared to all the other segments (p < 0.001). Movement 1 also had the lowest peak grip aperture (Fig 5B) compared to the other three movements (p < 0.001). The transport velocity for Movement 1 was similar to that of Movements 3 and 4 (p = 1.000).

Movement 2 required a change in grasp to a side grasp of the cup, and displayed a similar peak grip aperture to Movement 3 also requiring a side grasp (p = 0.959), but was significantly different from the top grasps (p < 0.05). Although Movements 2 and 3 had similar grip apertures, there were differences in the velocity profiles. Movement 2 had a lower and earlier peak hand velocity than Movement 3, and the earliest deceleration peak of all the reach-grasp segments. This slowed movement was also seen in the transport-release segment of Movement 2, which had the lowest peak hand velocity of all the transport-release segments. The percent-to-peak grip aperture occurred significantly earlier in the reach-grasp segment of Movement 2 compared to the other 3 movements (p < 0.01).

In contrast, the reach-grasp segment in Movement 3 had the highest peak hand velocity. The hand velocity for the transport-release segment of Movement 3 was not significantly different from Movement 1 (p = 1.000) and 4 (p = 0.137). Movement 4 had the largest peak grip aperture (p < 0.05). It otherwise showed similar characteristics in percent-to-peak grip aperture and percent-to-peak hand deceleration as Movement 1, which had the same grasp.

### Between-session repeatability

Both the Pasta Box Task and Cup Transfer Task presented mostly good repeatability (ICC > 0.75) for movement time, peak hand velocity, percent-to-peak hand velocity, peak grip aperture, percent-to-peak grip aperture, and percent-to-peak hand deceleration.

For the Pasta Box Task (Table 6), poor to moderate repeatability (ICC values of below 0.75) was found for only two measures: peak hand velocity and percent-to-peak hand velocity for the transport-release segment of Movement 1. For the Cup Transfer Task (Table 7), poor to moderate repeatability (ICC values of below 0.75) was found for six of the 36 measures: peak hand velocity for the transport-release segment of Movement 1, Movement 3, and Movement 4, as well as for percent-to-peak hand velocity, percent-to-peak grip aperture, and percent-to-peak hand deceleration for the reach-grasp segment of Movement 1.

**Table 6:** Pasta Box Task repeatability.

		**Movement** **time (sec)**			**Peak hand** **velocity (mm/s)**			**Percent-to-peak** **hand velocity (%)**		
		*ICC*	*SEM*	*MDC*	*ICC*	*SEM*	*MDC*	*ICC*	*SEM*	*MDC*
**Mvmt 1**	RG	0.84 (0.37–0.96)	0.035	0.098	0.89 (0.54–0.97)	38	106	0.90 (0.61–0.98)	1.4	3.8
	TR	0.81 (0.25–0.95)	0.046	0.128	*0*.*56 (-0*.*77–0*.89)*	56	156	*0*.*74 (-0*.*06–0*.*93)*	2.4	6.6
**Mvmt 2**	RG	0.88 (0.50–0.97)	0.030	0.083	0.79 (0.15–0.95)	64	177	**0.95 (0.79–0.99)**	1.6	4.4
	TR	0.86 (0.45–0.97)	0.057	0.159	**0.91 (0.64–0.98)**	30	84	0.83 (0.30–0.96)	2.0	5.5
**Mvmt 3**	RG	**0.93 (0.70–0.98)**	0.029	0.081	**0.93 (0.72–0.98)**	47	130	0.89 (0.57–0.97)	2.1	5.7
	TR	0.89 (0.56–0.97)	0.052	0.145	0.78 (0.10–0.94)	40	112	0.86 (0.45–0.97)	1.6	4.4
		**Peak grip** **aperture (mm)**			**Percent-to-peak** **grip aperture (%)**			**Percent-to-peak** **hand deceleration (%)**		
		*ICC*	*SEM*	*MDC*	*ICC*	*SEM*	*MDC*	*ICC*	*SEM*	*MDC*
**Mvmt 1**	RG	**0.95 (0.78–0.99)**	2	4	0.88 (0.50–0.97)	1.8	4.9	0.84 (0.35–0.96)	3.4	9.5
**Mvmt 2**	RG	**0.92 (0.68–0.98)**	2	4	**0.95 (0.81–0.99)**	1.0	2.7	0.80 (0.20–0.95)	2.6	7.2
**Mvmt 3**	RG	**0.95 (0.79–0.99)**	1	4	0.87 (0.47–0.97)	1.3	3.7	0.75 (0.01–0.94)	2.4	6.6

Pasta Box Task repeatability results. Repeatability analysis was performed for movements and segments separately, on movement time, peak hand velocity, percent-to-peak hand velocity, peak grip aperture, percent-to-peak grip aperture, and percent-to-peak hand deceleration. Repeatability measures include intra-class correlation (ICC) with corresponding 95% confidence intervals, standard error of measurement (SEM), and minimal detectable change (MDC). ICC values above 0.90 are presented in bold. ICC values below 0.75 are presented in italics. ICC values that failed the F-test (p > 0.05) are presented with an asterisks (*), indicating the validity of the ICC may be compromised for this result. Movements are: Movement 1 (Mvmt 1), Movement 2 (Mvmt 2), and Movement 3 (Mvmt 3); segments are: reach-grasp (RG) and transport-release (TR).

**Table 7:** Cup Transfer Task repeatability.

		**Movement** **time (sec)**			**Peak hand** **velocity (mm/s)**			**Percent-to-peak** **hand velocity (%)**		
		*ICC*	*SEM*	*MDC*	*ICC*	*SEM*	*MDC*	*ICC*	*SEM*	*MDC*
**Mvmt 1**	RG	0.83 (0.33–0.96)	0.032	0.089	0.77 (0.07–0.94)	64	176	*0*.*54 (-0*.*84–0*.89)*	4.6	12.6
	TR	0.85 (0.41–0.96)	0.042	0.116	*0*.*65 (-0*.*43–0*.91)*	50	139	0.82 (0.25–0.95)	0.9	2.5
**Mvmt 2**	RG	0.85 (0.40–0.96)	0.033	0.092	**0.96 (0.83–0.99**)	26	72	**0.93 (0.71–0.98)**	2.1	5.9
	TR	0.81 (0.22–0.95)	0.053	0.147	0.76 (0.03–0.94)	29	81	**0.91 (0.63–0.98)**	2.8	7.8
**Mvmt 3**	RG	0.85 (0.41–0.96)	0.045	0.125	0.76 (0.05–0.94)	60	166	**0.91 (0.62–0.98)**	2.1	5.9
	TR	0.76 (0.05–0.94)	0.068	0.189	*0*.*52 (-0*.*93–0*.88)*	25	68	0.85 (0.38–0.96)	0.8	2.1
**Mvmt 4**	RG	0.75 (0.01–0.94)	0.031	0.086	0.84 (0.35–0.96)	50	139	0.78 (0.10–0.95)	3.8	10.5
	TR	0.85 (0.38–0.96)	0.058	0.162	*0*.*64 (-0*.*44–0*.91)*	29	80	**0.99 (0.96–1.00)**	**0.7**	2.0
		**Peak grip** **aperture (mm)**			**Percent-to-peak** **grip aperture (%)**			**Percent-to-peak** **hand deceleration (%)**		
		*ICC*	*SEM*	*MDC*	*ICC*	*SEM*	*MDC*	*ICC*	*SEM*	*MDC*
**Mvmt 1**	RG	**0.97 (0.87–0.99)**	1	3	*0*.*60 (-0*.*61–0*.90)*	1.8	5.0	*0*.*68 (-0*.*30–0*.92)*	4.1	11.4
**Mvmt 2**	RG	**0.97 (0.86–0.99)**	1	3	0.79 (0.14–0.95)	2.1	5.9	0.86 (0.43–0.97)	2.8	7.9
**Mvmt 3**	RG	**0.97 (0.86–0.99)**	1	2	0.81 (0.24–0.95)	1.6	4.3	**0.95 (0.78–0.99)**	1.5	4.3
**Mvmt 4**	RG	**0.96 (0.83–0.99)**	2	4	0.86 (0.45–0.97)	2.2	6.0	0.84 (0.36–0.96)	5.2	14.5

Cup Transfer Task repeatability results. Repeatability analysis was performed, for movements and segments separately, on movement time, peak hand velocity, percent-to-peak hand velocity, peak grip aperture, percent-to-peak grip aperture, and percent-to-peak hand deceleration. Repeatability measures include intra-class correlation (ICC) with corresponding 95% confidence intervals, standard error of measurement (SEM), and minimal detectable change (MDC). ICC values above 0.90 are presented in bold. ICC values below 0.75 are presented in italics. ICC values that failed the F-test (p > 0.05) are presented with an asterisks (*), indicating the validity of the ICC may be compromised for this result. Movements are: Movement 1 (Mvmt 1), Movement 2 (Mvmt 2), Movement 3 (Mvmt 3), and Movement 4 (Mvmt 4); segments are: reach-grasp (RG) and transport-release (TR).

For the Pasta Box Task (Table 6), SEM values ranged from 1 to 7% of the average absolute measurement value across measures, and MDC ranged from 3 to 20% across measures. For the Cup Transfer Task (Table 7), SEM values ranged from 1 to 15% across measures and MDC ranged from 2 to 42% across measures.

## Discussion

The purpose of developing new standardized functional tasks representative of real-world ADLs was to create a meaningful assessment metric for clinical populations with upper limb impairments, that specifically focused on quantifying hand kinematics. Reach-grasp tasks have been shown to provide insights into altered motor control strategies in populations with impaired upper limb function [1,16,30,35–37]. The importance of involving objects in goal-directed tasks with a functional context has been previously demonstrated as resulting in smoother, faster, more preplanned movement compared to non-goal directed movement through space [38]. Using natural objects for completing a task and providing functional information on the objects, rather than simulated devices, is important to enhance functional performance in both normative and impaired populations [39]. Our tasks, designed to be consistent with these parameters and also the requirements of known clinical upper limb assessments [20,23–26,40,41], were relatively easy for non-disabled individuals to perform. However, since errors were made by participants, the tasks required some level of attention and concentration. This may be valuable for assessing clinical populations with not only motor difficulties but also motor planning impairments.

The tasks had specific movement sequences that were standardized, repeatable, of short duration, and consistently performed by individual participants. Other tasks used in literature have shown low within-participant variability [12,13,35], however typically using more constrained tasks not as representative of real-world object interactions. Within-participant variability is an important factor to assess as, for some clinical populations, increased variability in motor performance is a key indicator of poor motor skill, and may indicate the adoption of various strategies for accomplishing a task rather than converging on one strategy. For example, prosthesis users have been shown to have increased variability in upper limb angular kinematics, as reflected by increased average standard deviation [36], whereas more skilled prosthesis users have less deviation in end-point kinematic profiles from non-disabled movement patterns [42]. The measurement of variability may play an important role given that it has been shown in the occupational literature that both kinematic compensation and motor variability are associated with musculoskeletal pain [43]. The inclusion of mean participant standard deviation as a measure of within-participant variability in this normative data set will allow comparison in future study of impaired populations. The between-session repeatability of the task was also found to be good for 55 out of 63 parameters, which is a prerequisite prior to investigating sensitivity to change in clinical populations.

The task design with specific sequencing allowed for segmentation of movements into the crucial phases of reaching and grasping, and transporting and releasing objects within the same task. This allows examination of discrete characteristics of hand movement pertaining to hand trajectory, hand velocity, and grip aperture for each of these phases. This is important as many clinical populations will have impaired dexterity impacting grasp, which has been extensively investigated [30,44]; however, valuable information can also be obtained by examining control of the hand during transport, such as grip modulation. In addition, grasp features are known to be affected by the task goal and setting [19]; therefore, it is most ecologically valid to use tasks that not only reach and grasp but involve a logical next step of movement and placement of the object.

The influence of an object’s intrinsic and extrinsic parameters on hand kinematics was consistent with prior literature. Location of the object influenced several parameters, particularly the first movement of the Pasta Box Task where the box of pasta was not within the direct field of view and required a turn of the body and the head for the grasp. This misalignment of the body space to the visual space has been shown to increase the latency of the movement towards a target and decrease accuracy [45,46]. This first movement also required the arm to move multiple degrees of freedom across several planes (i.e., sagittal, transverse, and coronal planes) to complete the movement accurately. Therefore, a greater deceleration phase was necessary, evidenced by the hand velocity peaks occurring earlier than for other movements. This aligns with previous research by Fisk & Goodale who found hand velocity peaks to occur roughly around one third of the movement for lateral reaches [45], compared to studies that restricted reaching tasks to single plane movement and reported more symmetrical hand velocity profiles [47].

The location of the cups in the task space and the required grasp conformation also influenced reaching strategies. The first reach-grasp of the Cup Transfer Task showed the smallest grip aperture, suggesting confidence with the upcoming grasp of the top of the cup, but a slowed velocity of the reach likely due to the short distance. The two cylindrical side grasps showed similar grip apertures, but were different in movement strategies in that the first cylindrical side grasp showed several features suggesting it was the reach with the highest perceived risk, with lower, earlier peak velocity and the earliest deceleration. This is consistent with previous studies where hand velocity was lower during the reach when the task following the approach required precision [48,49]. Three of the transport release segments showed peak velocities occurring no later than 25% of the movement, indicating that movement of the compliant cup with risk of spillage was potentially challenging. This is consistent with Butler et al. who found lower percent-to-peak hand velocity values for the segment of their task where the performer had to bring a cup to their mouth, suggesting that this movement was riskier and required more conservative control strategies [35].

Both tasks involved obstacle avoidance; in the vertical plane for every movement of the cups, and in the horizontal plane for the second movement of the box of pasta. As previously shown by Chapman & Goodale, obstacles change the spatiotemporal characteristics of hand movement by increasing movement time and decreasing peak hand velocity [50]. This effect is even greater when obstacles are closer to the performer and on the side of the reaching arm [50]. These differences in spatiotemporal characteristics may have been amplified in our task since these trends were observed during the transport-release segment as opposed to the reach-grasp segment, where moving the object adds a further level of uncertainty.

The challenges presented by these varied intrinsic and extrinsic properties is expected to result in significant performance differences in conditions with impaired hand sensation and impaired upper limb function. Reaching and grasping are functionally linked to the specific task, with the characteristics of the object determining the relative timing of peak grip aperture and peak hand deceleration [51]. Abnormalities in these features resulting in decoupling of reach and grasp have been shown in prosthesis users [18], cerebellar lesions [37], spinal cord injury [2] and in stroke populations [52], suggesting that this type of kinematic analysis could have applicability to multiple populations with upper limb impairment.

### Limitations and future work

The assessment of normative hand movement characteristics demonstrated consistent trends across varying task challenges. The limitations of the current study include the assessment of only between-session repeatability, not the repeatability among different test administrators and study sites. Further study of inter-rater repeatability will assist with determining reproducibility of the task assessment. Considering that the presented normative data set establishes an ideal young adult performance standard, further work may focus on establishing differences between the sexes or with aging cohorts to obtain a fully comparative data set for populations with impairment. Finally, future work will also test the application of this methodology in populations with upper limb impairments, and validate the measures against other clinically validated hand outcome assessments in these populations.

## Conclusion

Standardized upper limb functional tasks which mimic ADLs and incorporate elements of risk and accuracy, lateral reaches, reaches crossing the body’s midline, objects of different shapes and sizes, and different grasp patterns to assess hand movements were developed. A normative dataset for hand movement was created based on non-disabled performance characterizing hand trajectory, hand velocity, and grip aperture features for reach-grasp and transport-release segments of the movements. These features verified that the tasks challenged a variety of motor control strategies, and these unique movement characteristics were reflected in the quantitative results while being highly consistent within-performers. In addition to the low within-participant and between-participant variability for these complex tasks, a repeatability analysis showed that this novel assessment approach has good between-session repeatability. This assessment promises to be a valuable tool for future research in populations with upper limb impairments.

## Supporting information

S1 Text Pasta Box Task. (PDF)

S2 Text Cup Transfer Task. (PDF)

S1 Table Supplementary data file. (XLSX)
