# Supplementary material for: Characterization of normative hand movements during two functional upper limb tasks
Source: PLoS One. 2018 Jun 21;13(6):e0199549. doi: 10.1371/journal.pone.0199549 (PMC6013217; doi:10.1371/journal.pone.0199549)
Supplement: S1 Text — (PDF) [file pone.0199549.s001.pdf]

Pasta Box Transfer Task

Detailed Task Protocol

This document outlines the set-up and task protocol for the Pasta Box Task.

Task Design.. 2
Task Set-Up 3
Task Overview 5
Task Instructions to Participant 6
Performance Metrics... 7

Last Updated: December 15, 2017

Task Design

This task assesses the ability to reach, grasp, transport, and release a rectangular deformable object (box of pasta) within a standardized table/shelf height set up, at different levels and across midline (Fig 1).

- **Pasta box** dimensions: 7 x 3.5 x 1.5 inches; weight 225 grams CAN (i.e. "Kraft Dinner"); 7.25 ounces/206g US (i.e. "Kraft macaroni and cheese")
- **Shelving unit:** counter height table (36 inch); middle shelf 7 inches from counter top; high shelf 12 inches from counter top (see Appendix A). Neutral eye position marker (reflective marker on orange paper), 18.5 inches from counter top on the front of the middle shelf. Entire shelving unit is 9 inches back from the front edge of the table.
- **Cart** or small table placed to right of shelving unit: 30 inch height
- **Motion capture markers:** placed on table, cart and pasta box as outlined in Task Set-Up

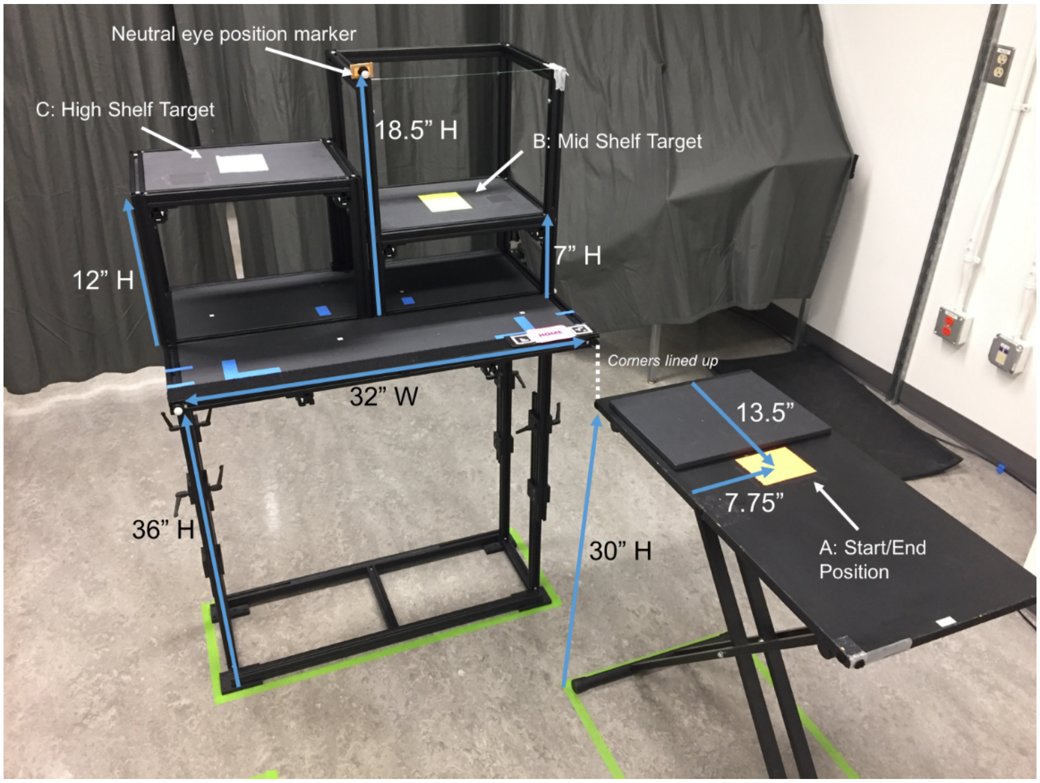

Figure 1. Set up for Pasta Box Transfer Task

Task Set-Up

***NOTE: The following sections are written for a person using their RIGHT hand or terminal device. For testing the LEFT hand or terminal device, positions should be TRANSPOSED.

The shelving unit consists of a counter height platform (36 in high), with a RIGHT side middle shelf 7 inches from counter height (Figure 1, position B) and a LEFT sided high shelf 12 inches from the counter height (Figure 1, position C). Each “Target” position is a 3.5” x 4.5” rectangle. The center of the mid shelf target is 8 inches right of the midline of the able and 15 inches from the front edge of the table. The center of the high shelf target is 8 inches left of the midline of the table and 15 inches from the front edge of the table (Figure 2).

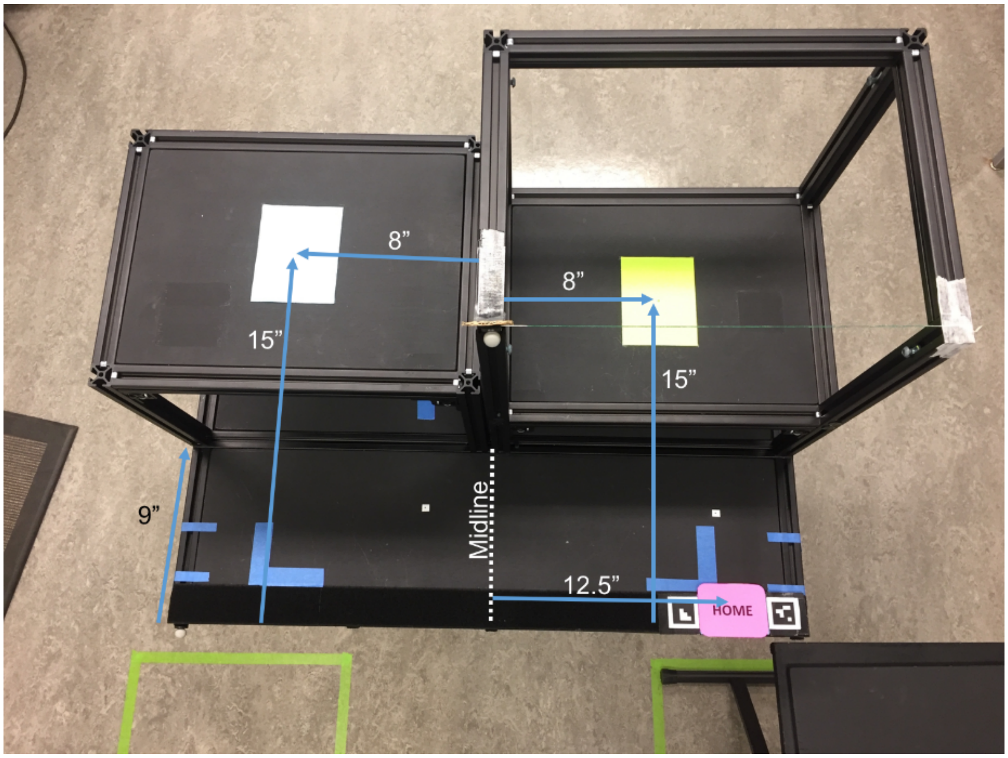

Figure 2. Top View of Set up for Pasta Box Transfer Task

The RIGHT corner of the shelving unit has a HOME sticker (3.25” x 2.5”) at the front edge, with its center 12.5 inches right of the midline of the table. The top middle of the shelving unit has a NEUTRAL orange sticker or marker for the participant to fixate on at the beginning and the end of the trial, 18.5 inches from counter height.

The pasta box is placed in a vertical orientation (on its base) on a table 30 inches high to the RIGHT of the shelving unit (Figure 1, position A: Start position). The table is placed so that the LEFT front corner of the table is immediately adjacent to the shelving unit at the RIGHT corner edge (near the HOME position). Relative to the LEFT front corner of the table, the center of the START position target is 13.5 inches to the right and 7.75 inches towards the middle of the table (Figure 1).

The participant stands so that their testing hand/prosthetic terminal device is resting on the standardized HOME position, placed on the RIGHT corner of the counter height shelf, with eyes fixated at neutral, at the start and end of every trial.

Motion capture markers should be placed on the base of the shelving cart, the side table, and the pasta box as indicated in Figure 3.

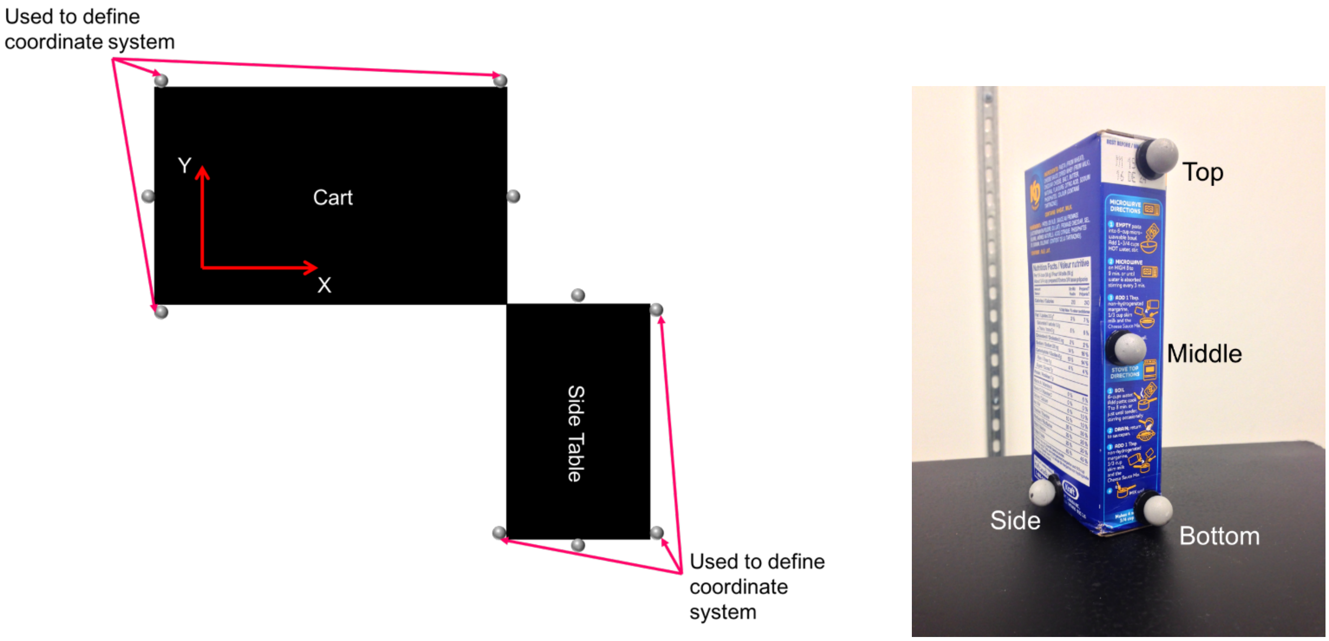

Figure 3. Location of reflective markers for tables (left) and on Pasta Box (right)

Refer to specific protocols for motion capture and eye tracking calibration and data collection process.

Task Overview

The participant will be asked to move the pasta box in 3 discrete transport movements. In-between each grasp-transport-release movement, the hand/terminal device will return to the HOME position.

1. The participant will move the pasta box from the start position to the middle shelf, place the box on its base in the defined target on the shelf, and then return hand to HOME.
2. They will grasp the box from the middle shelf and move it to the high shelf on the LEFT and place it on its base on the target square, and return hand to HOME.
3. Finally, they will move the box from the high LEFT shelf back down to the original starting position and release it on its base, and return hand to HOME.

Task Instructions to Participant

(*Demonstrate the task while explaining*)

Please stand comfortably in front of the cart, so that you can reach the target areas where you will be moving the pasta box. Your body should be centered to the task, and you should try not to take steps to move your body but you can shift your weight. Try to move as naturally as you would in your own environment performing similar tasks.

You will start every trial with the RIGHT hand at the home position and your eyes fixated on NEUTRAL. There are 3 separate movements of the pasta box that we want you perform.

When prompted to start, you will grasp the side of the pasta box, move it up to the middle shelf in the defined **green target** and place it on its base, release, then place your hand back on the HOME sticker.

You will then reach for the box on the middle shelf, grasp it, and bring it over to the top shelf on the left, and release it on its base in the **blue target**. You will then move your hand back to HOME.

You will then reach for the box on the top shelf and move the box back down to the START position on the table to your RIGHT. You will then place your hand back on the home position and look at the neutral eye position to end the trial.

If you drop the box or bump it on anything, continue the movement sequence from where you left off and finish the motion. Make sure that the box is always released so that it is on its base, and not on its side.

I will now demonstrate what the execution of the task should look like *(DEMO full task).*

Perform these movements at a comfortable pace that will allow you to be as accurate as possible, without dropping the box or hitting the edges of the shelving. You will be timed, and errors such as dropping the box or not placing it on the correct edge will be recorded.

Do you have any questions about the task?

You can now practice performing the task. *(Allow practice for minimum 1 trial, or until participant is comfortable with the task)*

(When ready to begin the trials): You will be prompted by the researcher saying "**eyes on neutral, hand on home"** at the start of each trial, then a **"beep"** is the signal that you can start the trial.

(Follow experimental protocol for # trials)

Performance Metrics

- Time for total task (start and finish at HOME)
- **Errors**: record number and type of errors
  - Dropped box
  - Box grasped incorrectly (i.e. Box was grasped from the top, rather than on the thin side)
  - Box incorrectly placed (i.e. Box was placed on its side versus on its base)
  - Participant hit the frames of cart with the box
  - Incorrect task sequence (i.e. Participant did not go home before moving from position B to position C)
  - Box placed outside of the target (i.e. Pasta box rests half on the target, half off the target)

Appendix A. Task Cart Design

The Task Cart has been designed for use with the pasta box, cup transfer, shape sorting and cup pouring tasks. The design is modular to allow rapid adjustments as needed.

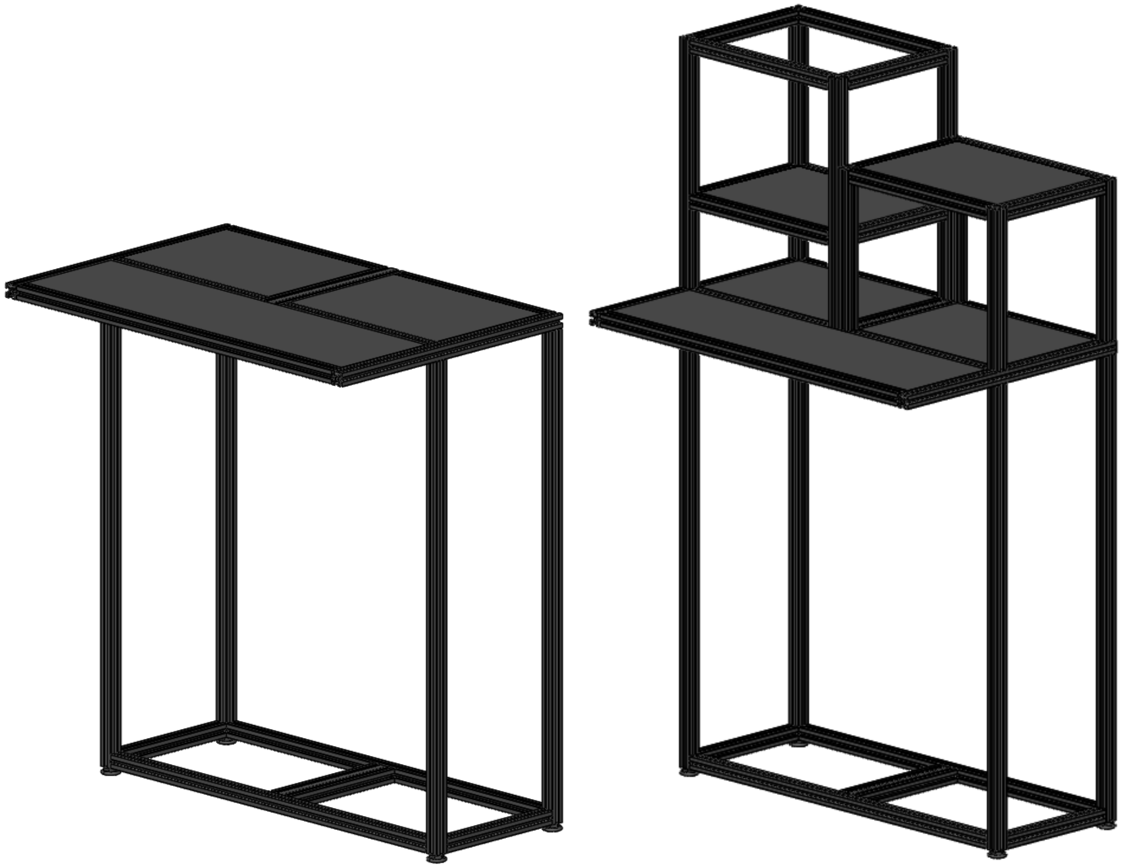

(a) (b)

Fig. A.1 Task cart (a) base, and (b) with shelves

There are several key design features of the task cart that make it useful for the designed tasks.

Shelves are designed to be removed and attached to quickly transition between tasks, while being secure during use. The end of each shelf leg contains a fastener that allows it to slide along the beams on the cart surface, enabling the shelves to slide in and out of the back of the cart. The shelves are secured in place by inserting end caps into the back of the cart beams by hand.

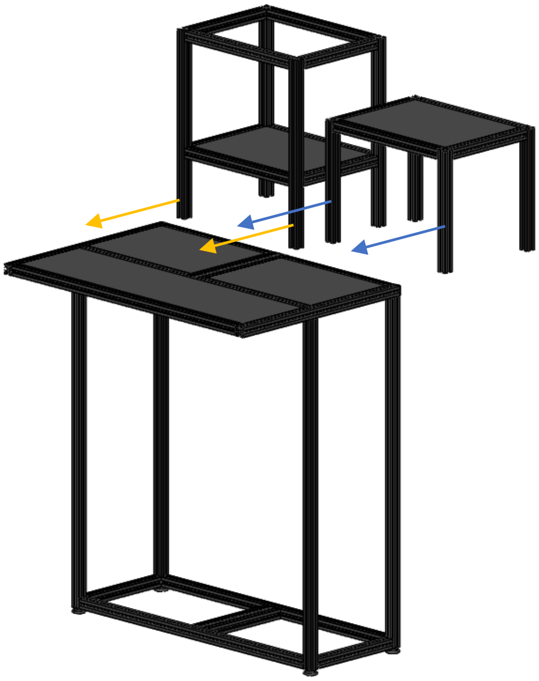

Fig. A.2 Cart with shelves being slid in place from back

The top beam on the tall shelf can be exchanged for a wire, in case the beam interferes with eye fixation areas for the task. There are holes in the shelves to facilitate ease of wire attachment.

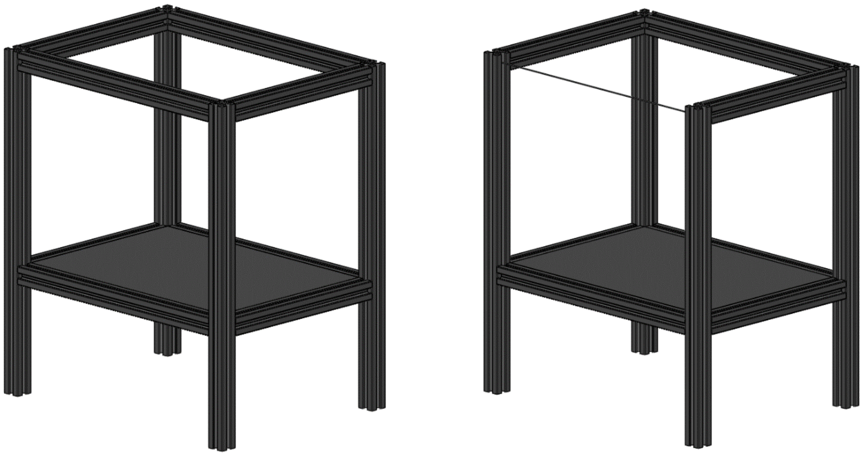

(a) (b)

Fig. A.3 Tall shelf with options (a) beam, and (b) wire

The cart and shelves have been designed using [80/20 slotted framing] and can be ordered directly through [Rocky Mountain Motion Control] by providing them with the following design number: Q000002492. Alternatively, the shelves may be purchased alone.

Drawings are provided below. A SolidWorks model and assembly instructions are available; if desired please contact the BLINC lab.

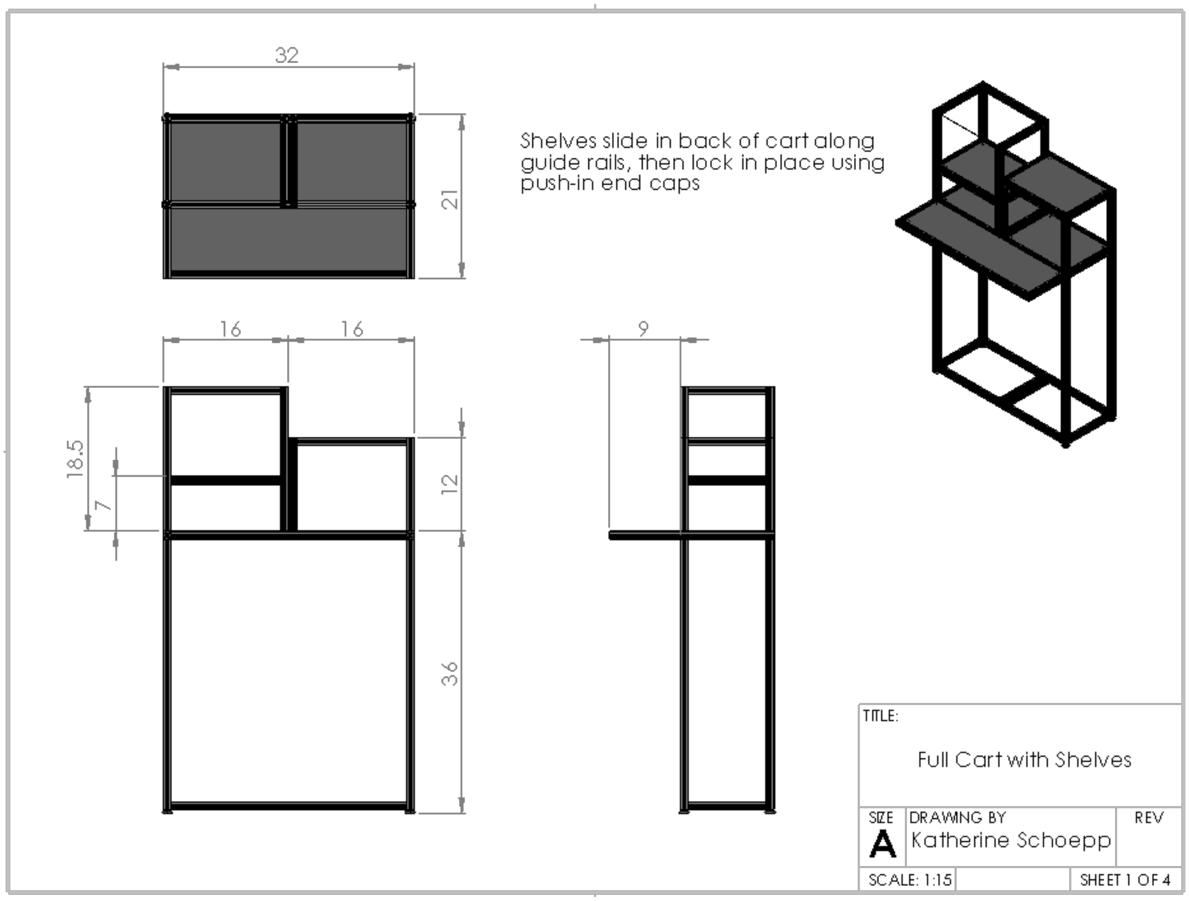

(a)

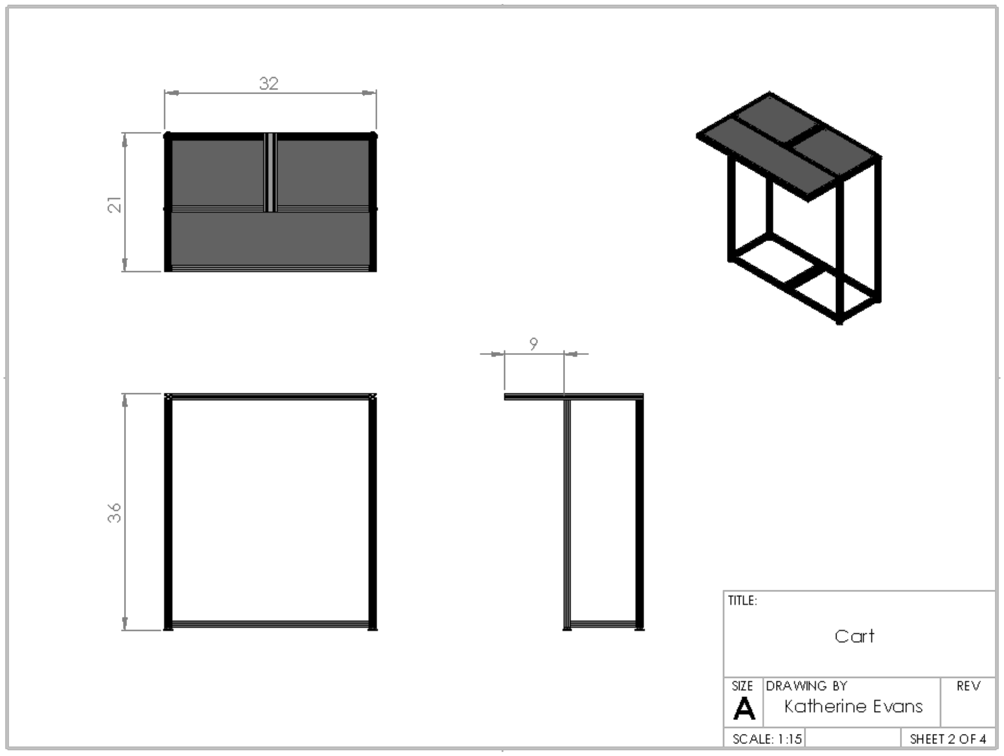
32
21
9
36
TITLE:
Cart
SIZE
A
DRAWING BY
Katherine Evans
REV
SCALE: 1:15
SHEET 2 OF 4

(b)

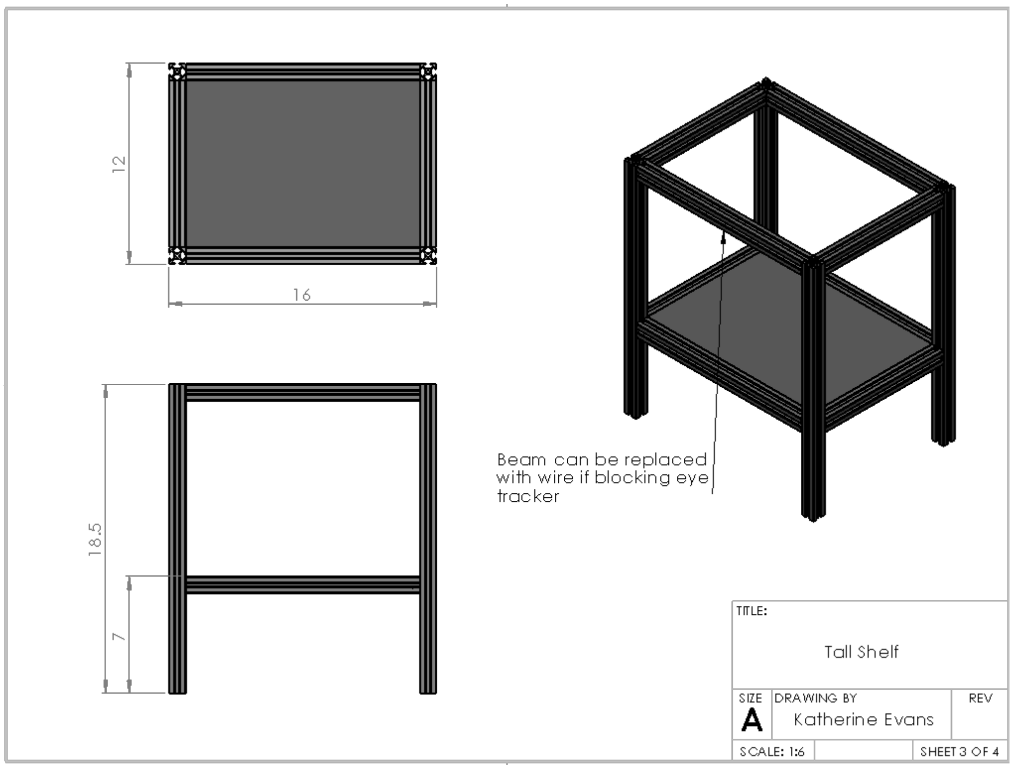
12
16
18.5
7
Beam can be replaced with wire if blocking eye tracker
TITLE:
Tall Shelf
SIZE
A
DRAWING BY
Katherine Evans
REV
SCALE: 1:6
SHEET 3 OF 4

(c)

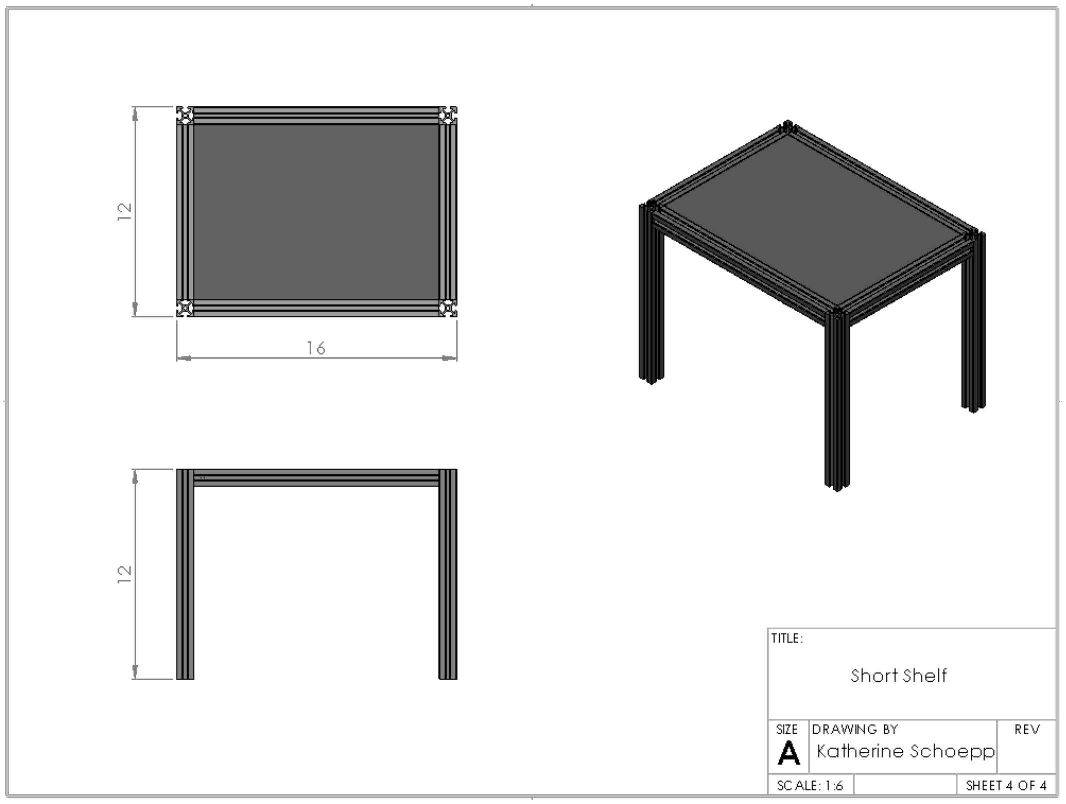

(d)

Fig. A.4 Drawings for designed task cart, (a) full cart with shelves, (b) cart base, (c) tall shelf, and (d) short shelf
