# Supplementary material for: Characterization of normative hand movements during two functional upper limb tasks
Source: PLoS One. 2018 Jun 21;13(6):e0199549. doi: 10.1371/journal.pone.0199549 (PMC6013217; doi:10.1371/journal.pone.0199549)
Supplement: S2 Text — (PDF) [file pone.0199549.s002.pdf]

Cup Transfer Task

Detailed Task Protocol

This document outlines the set-up and task protocol for the Cup Transfer Task.

Task Design 2
Task Set-Up 3
Task Overview 5
Task Instructions to Participant 6
Performance Metrics 7

Last Updated: March 15, 2018

Task Design

This task assesses the ability to grasp, modulate grasp force (with risk), transport, and release a deformable object across midline in a confined space with pre-determined placement.

- **Box design**: Interior dimensions 30 in. wide, 14 in. deep, 3 in. high box edges, central divider 6 in. high (Figure 1, Appendix A).
- **Targets:** Two defined start and stop targets marked on each side of the box (blue for FAR cup, green for NEAR cup). The centers of the targets are 3 inches from the nearest two box edges (Figure 2).
- **Material:** Two standard 5 oz. Wax Treated Paper Cold Cups (58PATH, Dixie Consumer Products, LLC) filled with beads (Soft Plastic Pellets (A4155 - Phase 2, Patterson Medical Holdings, Inc.) to a weight of 85 grams / 3 ounces (including the weight of the cup).
- **Motion Capture reflective markers:** Placed on the back two corners of the box, and the back top of the middle divider. Each cup should also have a marker as indicated (Figure 3). The table should have 5 markers on the base, as per the pasta box task (refer to Pasta Box Protocol).
- **Table:** Standard counter height (36 inches); box placed 2.5 inches back from the near edge. HOME hand position is on the front edge of the table, with its center 12.5 inches to the right from the midline of the table. "Neutral" eye position marker at back edge of divider, 16.5 inches from the front edge of the table (Figure 1).

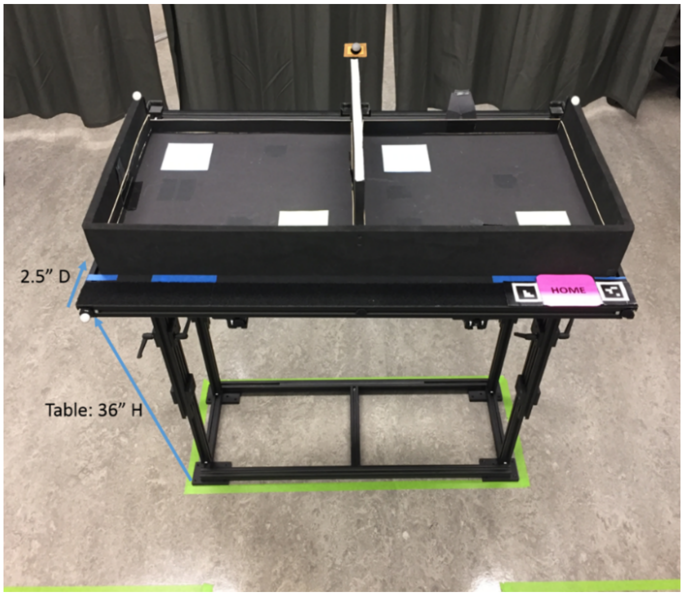

Figure 1. Cup Transfer Task Set Up

Task Set-Up

***NOTE: The following sections are written for a person using their RIGHT hand or terminal device. For testing the LEFT hand or terminal device, positions should be TRANSPOSED.

The box is placed on a standard counter height table (36 inches high), 2.5 inches back from the front edge, and aligned with the center of the table. A HOME sticker (3.25" x 2.5") is placed on the right-hand side flush with the front edge of the cart, and its middle point 12.5 inches right of the midline of the table. This will be the start and stop position for the hand / terminal device being tested.

A NEUTRAL orange sticker or marker should be placed at the back edge of the divider for the participant to fixate on at the beginning and the end of the trial. Reflective markers should be placed as indicated in Task Design and shown in Figure 2. In addition, both cups require a marker affixed as per Figure 3.

Within the box, the cups are placed on the side of the testing arm (RIGHT). As per Figure 2, cups are placed in two pre-determined positions, Far (blue targets) and Near (green targets).

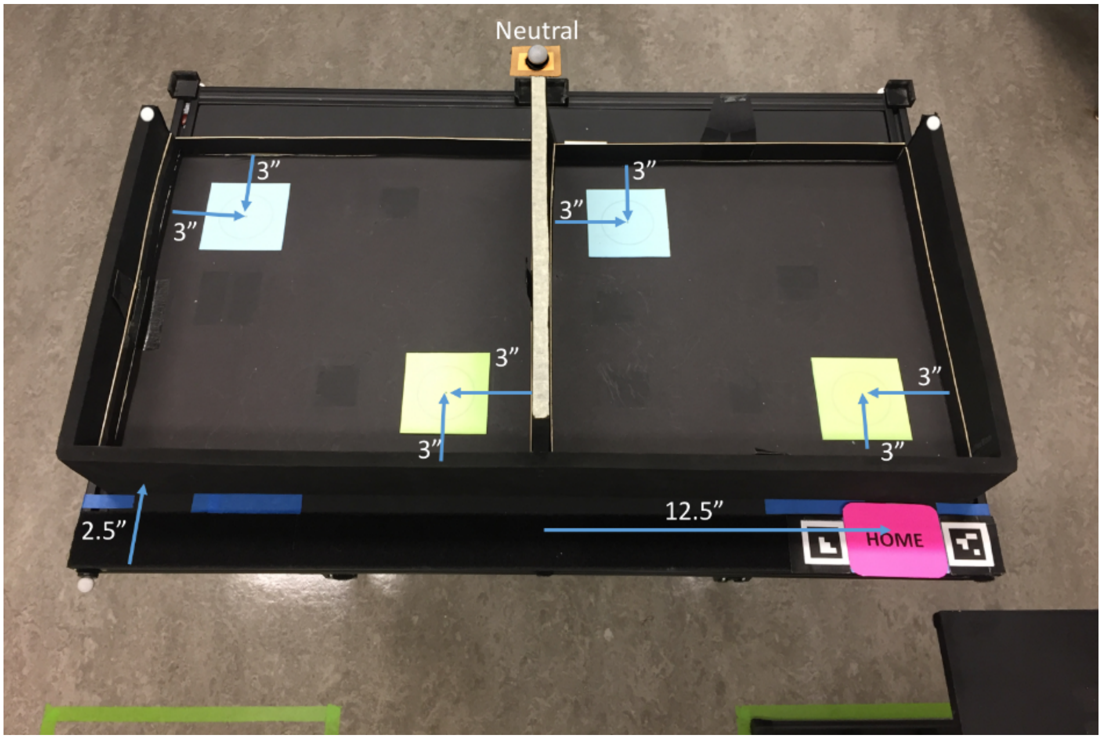

Figure 2. Interior Box Set Up for Cup Task

- **Far position** – The center of the starting FAR target is 3 inches from the back edge of the box and from the partition; the target is colored blue. The corresponding FAR target on opposite side of box (back left corner of left side of box) is also marked blue, its center is 3 inches from back and left sides of box. The blue cup has a colored blue stripe through its center (made with permanent marker).
- **Near position** – The center of the starting NEAR target is 3 inches from the right and the front edges of the box; the target is colored green. The corresponding center of the NEAR target on the opposite side of the box is 3 inches from the divider and the front edge of the box. The green cup has a colored green stripe along the top rim of the cup (made with permanent marker).

Cups are prefilled with the bead pellets, but not overflowing (Figure 3).

- The cups should be prefilled with bead pellets. Place the cup on a weigh scale, and add beads until a weight of 85 g / 3 ounces is reached. At the start of the trial, the cups are placed in corresponding targets on the RIGHT side of the box (side of the testing arm). Note that if a cup is crushed / beads are spilled during the task, a new cup should be inserted for the next trial.

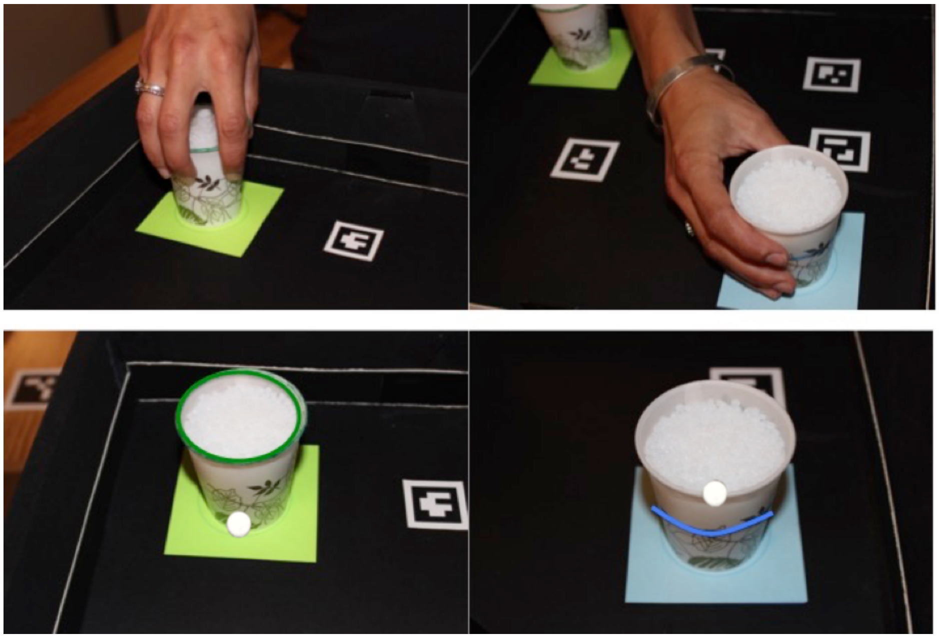

Figure 3. Correct Grasp Patterns and Marker Placement

Refer to specific protocols for motion capture and eye tracking calibration and data collection process.

Task Overview

1. The participant is asked to stand at a comfortable distance away from the table, centered to the task, so that they can reach the cups.
2. They start with their testing hand/prosthetic terminal device on the HOME sticker, and eyes looking at NEUTRAL.
3. When the experimenter instructs the participant (either by saying start, or prompted by a computer beep), they will pick up the NEAR cup first, move it over to the equivalent NEAR target on the left, then return to pick up the FAR cup, and move it to the equivalent FAR position on the left. They will then bring their hand/terminal device back to HOME.
4. They will then reach to the FAR cup on the left and return it to the FAR position on the right side of the divider, and then move the NEAR cup on the left back to the NEAR position on the right side of the divider, and then bring their hand/terminal device back to HOME.
5. Note that the NEAR cup requires a "top" grasp (as prompted by the green rim), and the FAR cup will require a "side" grasp (as prompted by the blue band around the cup), as per Figure 3.
6. One task demonstration by the experimenter and a minimum of 1 practice trial for the participant, is recommended. The number of repetitions for trials is determined based on the specific experimental protocol.

Task Instructions to Participant

(*Demonstrate the task while explaining*)

Please stand comfortably in front of the table, so that you can reach the cups. Your body should be centered to the task, and you should try not to take steps to move your body but you can shift your weight. Try to move as naturally as you would in your own environment performing similar tasks.

You will start every trial with your RIGHT hand/terminal device on the HOME area, and your eyes fixated on NEUTRAL. When prompted to start, you will reach for the NEAR cup with the GREEN rim, grasp it from the top, and move it OVER the partition, and place the cup in the GREEN target area on the other side of the partition. Try not to touch the partition, and you must clear the cup OVER the partition, not around the front. Be sure to place the cup on the target area. Be careful to not drop the cup or spill the beads inside.

Once the NEAR cup has been placed in the target area, move to the FAR cup and grasp it from the SIDE. Lift the cup over the divider, and place it on the BLUE target. After placing this cup on the target area, return your hand/terminal device to the HOME position.

You will then do the reverse of these movements: Reach over and grasp the FAR cup with a side grasp, and bring it back over the divider to the BLUE starting position. Then reach and grasp the NEAR cup with a top grasp, and move it back to the GREEN start position, then return your hand to HOME, with eyes on neutral.

I will now demonstrate what the execution of the task should look like *(DEMO full task).*

Perform this task at a comfortable pace that allows you to be as accurate as possible, without spilling any beads. You will be timed, and the experimenter will record errors such as dropping the cup, spilling the beads, or not placing the cup properly on the target. If you squish the cup or spill the beads, continue the task to the best of your ability.

Do you have any questions about the task?

You can now practice performing the task. (Allow practice for minimum 1 trial, or until participant is comfortable with the task)

(When ready to begin the trials): You will be prompted by the researcher saying "**eyes on neutral, hand on home"** at the start of each trial, then a **"beep"** is the signal that you can start the trial.

(*Follow experimental protocol for # trials*)

Performance Metrics

- Time for total task (start to finish at HOME)
- Number of **Errors**: record number and type of errors:
  - Squished cup
  - Weight of spilled beads: reweigh cup at end of each trial if spillage
  - Dropped cup (will likely need to redo trial, but record error and phase of error – during grasp, transport or release)
  - Incorrectly grasped cup (note grasps may be limited by type of prosthetic terminal device; in this case note type of grasp used).

Note regarding cup compression:

- The gradual force applied to spill beads in an unused cup is approximately 3.9 ± 0.5 N using a side grip, and 4.6 ± 0.6 N using a top grip. This force decreases significantly once the cup rim has cracked (to 3.6 ± 0.4 N and 3.3 ± 0.4 N, respectively), so a new cup should be used for each trial.

Appendix A. Cup Transfer Task Tray Design

The task tray is constructed using a stiff material (so that it does not collapse with pressure), such as Soleflex. The back walls are removable and are held in place using three clamps, 3D printed using a rigid material such as PLA. Solid models of these clamps may be requested. Refer to figures below for dimensions.

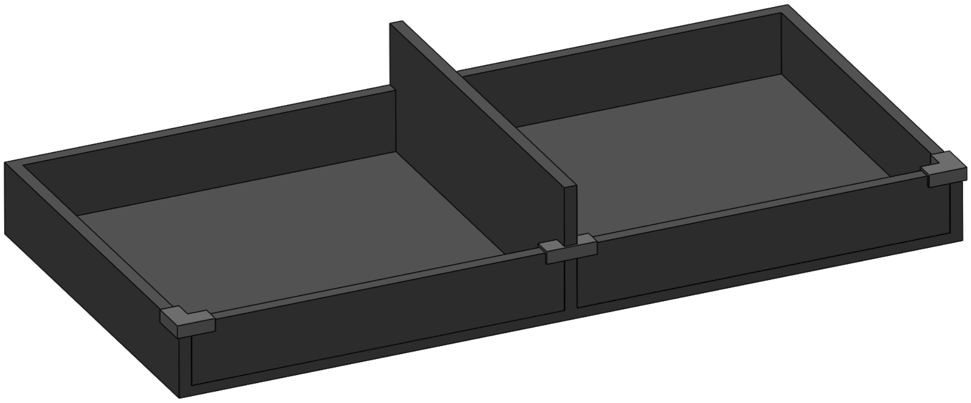

Figure A.1 Back view of tray assembly

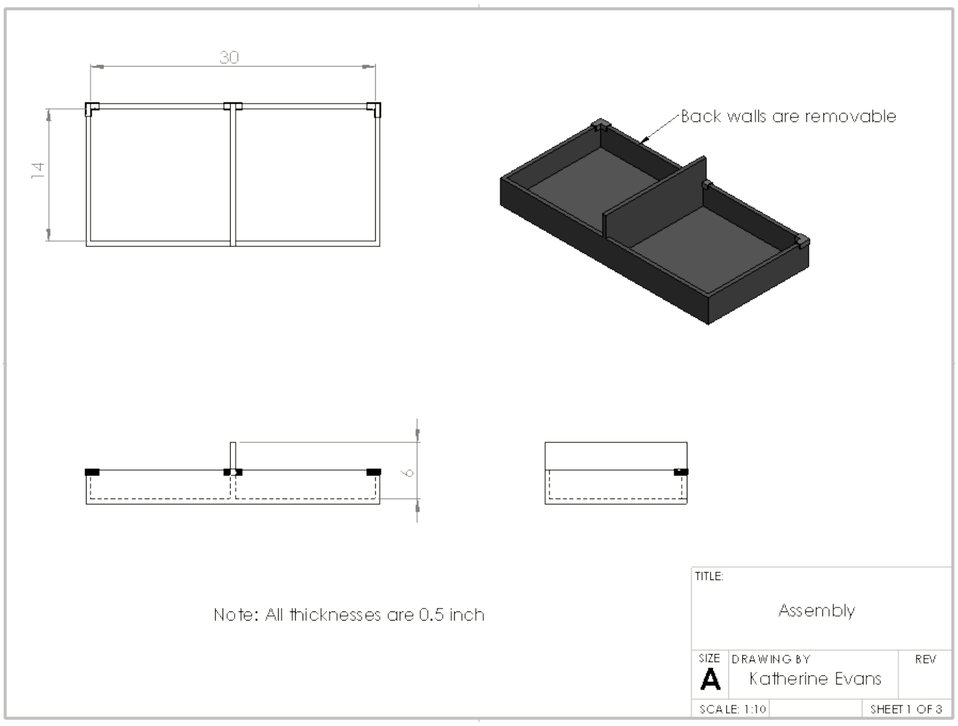

Figure A.2 Assembly of tray components

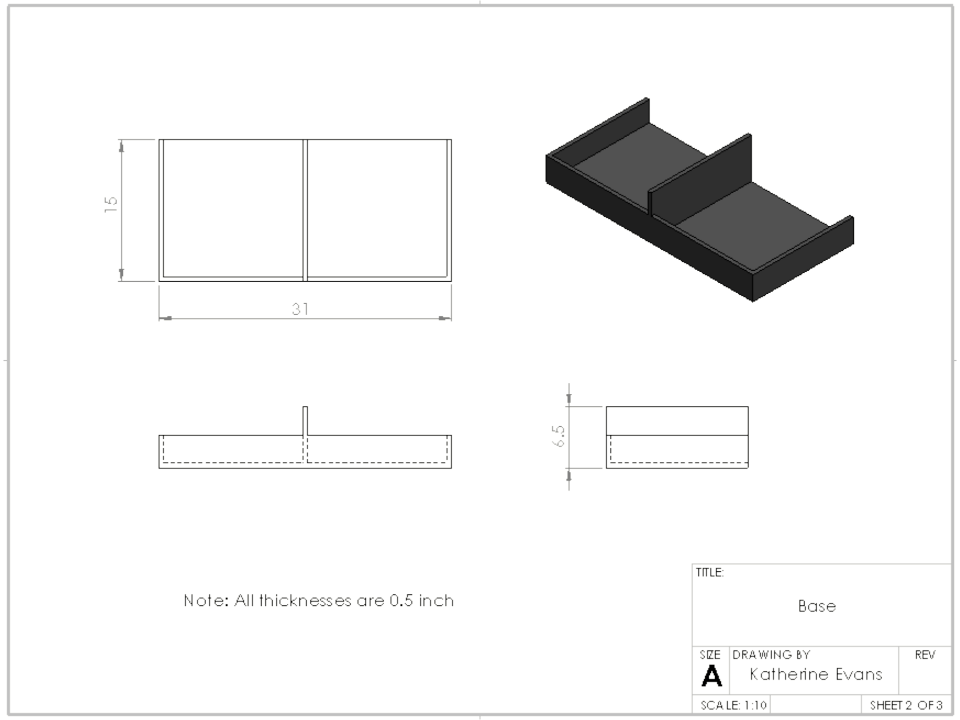

Figure A.3 Base dimensions

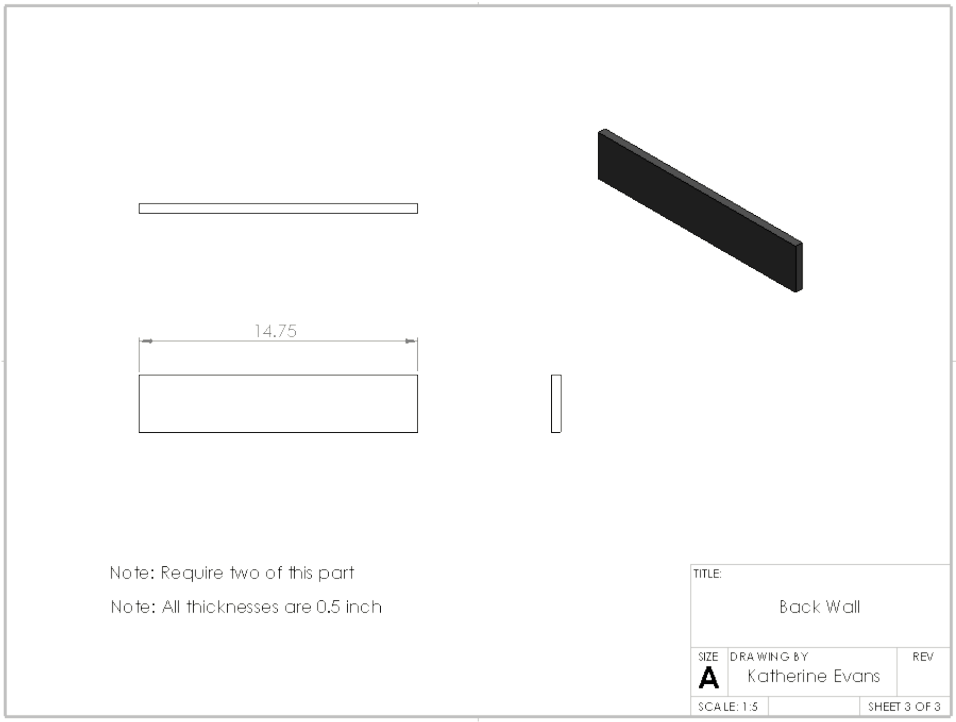

Figure A.4 Back wall
